# Supplementary material for: Hybrid Models and Biological Model Reduction with PyDSTool
Source: PLoS Comput Biol. 2012 Aug 9;8(8):e1002628. doi: 10.1371/journal.pcbi.1002628 (PMC3415397; doi:10.1371/journal.pcbi.1002628)
Supplement: Text S4 — Complete source code for the PyDSTool package (version 0.88.120504). Includes API documentation and help files linking to web pages. This file is identical to the current public release on Sourceforge.net. (ZIP) [file pcbi.1002628.s004.zip › PyDSTool/html/PyDSTool.Toolbox.dssrt-pysrc.html]

xml version="1.0" encoding="ascii"?


PyDSTool.Toolbox.dssrt


| Home | Trees | Indices | Help | PyDSTool |
| --- | --- | --- | --- | --- |

Package PyDSTool ::
Package Toolbox ::
Module dssrt

[hide private]

[frames] | no frames]

# Source Code for Module PyDSTool.Toolbox.dssrt

```
   1  """Implementation of dominant scale analysis techniques for python. 
   2   
   3  Loosely based on Matlab tool DSSRT. This module is incomplete and is 
   4  still a work in progress! See my publications on this subject for further 
   5  details. 
   6   
   7  Robert Clewley, 2009 
   8   
   9  """ 
  10  from __future__ import division 
  11   
  12  from PyDSTool import common 
  13  from PyDSTool import utils 
  14  from PyDSTool import HybridModel, NonHybridModel, Interval 
  15  from PyDSTool import parseUtils 
  16  from PyDSTool import Symbolic, Vode_ODEsystem 
  17  from PyDSTool.Trajectory import numeric_to_traj 
  18  from PyDSTool.Points import Point, Pointset, pointsToPointset 
  19  from PyDSTool.errors import * 
  20   
  21  import numpy as np 
  22  import scipy as sp 
  23  import copy 
  24  import cStringIO, operator 
  25   
  26  ############# 
  27   
  28  _classes = ['epoch', 'regime', 'dssrt_assistant', 'domscales', 
  29              'Scorer', 'EpochSeqScorer', 'VarAlphabet'] 
  30   
  31  _predicates = ['is_active', 'is_inactive', 'is_modulatory', 
  32                 'is_most_dominant', 'is_fast', 'is_slow', 'is_order1', 
  33                 'join_actives', 'leave_actives', 'become_most_dominant', 
  34                 'join_fast', 'join_slow', 'leave_fast', 'leave_slow'] 
  35   
  36  _functions = ['find_epoch_period', 'transition_psi', 'transition_tau', 
  37                'get_infs', 'get_taus', 'split_pts', 'define_psi_events', 
  38                'normalized_psis', 'define_tau_events', 'find_regime_transition', 
  39                'find_ep_ix', 'show_epochs', 'plot_psis', 'indent', 
  40                'swdist', 'jaro', 'comp_seqs', 'editdist_edits', 
  41                'MATCH', 'MISMATCH', 'APPROX', 'CLOSE', 'VCLOSE', 
  42                'FAR', 'VFAR', 'GAP', 'get_symbol_sequence', 'tabulate_epoch_seqs'] 
  43   
  44  __all__ = _classes + _functions + _predicates 
  45   
  46  ############# 
  47   
  48   


49 -class join_actives(common.predicate):


50      name = 'join_active' 
  51   


52 -    def precondition(self, epochs):


53          res = self.subject not in epochs[0].actives 
  54          self.record = (self.name, self.subject, res) 
  55          return res

  56   


57 -    def evaluate(self, epoch):


58          return self.subject in epoch.actives

  59   
  60   


61 -class leave_actives(common.predicate):


62      name = 'leave_actives' 
  63   


64 -    def precondition(self, epochs):


65          res = self.subject in epochs[0].actives 
  66          self.record = (self.name, self.subject, res) 
  67          return res

  68   


69 -    def evaluate(self, epoch):


70          return self.subject not in epoch.actives

  71   
  72   


73 -class become_most_dominant(common.predicate):


74      name = 'become_most_dominant' 
  75   


76 -    def precondition(self, epochs):


77          res = self.subject != epochs[0].actives[0] 
  78          self.record = (self.name, self.subject, res) 
  79          return res

  80   


81 -    def evaluate(self, epoch):


82          return self.subject == epoch.actives[0]

  83   
  84   


85 -class join_fast(common.predicate):


86      name = 'join_fast' 
  87   


88 -    def precondition(self, epochs):


89          res = self.subject not in epochs[0].fast 
  90          self.record = (self.name, self.subject, res) 
  91          return res

  92   


93 -    def evaluate(self, epoch):


94          return self.subject in epoch.fast

  95   
  96   


97 -class join_slow(common.predicate):


98      name = 'join_slow' 
  99   


100 -    def precondition(self, epochs):


101          res = self.subject not in epochs[0].slow 
 102          self.record = (self.name, self.subject, res) 
 103          return res

 104   


105 -    def evaluate(self, epoch):


106          return self.subject in epoch.slow

 107   
 108   


109 -class leave_fast(common.predicate):


110      name = 'leave_fast' 
 111   


112 -    def precondition(self, epochs):


113          res = self.subject in epochs[0].fast 
 114          self.record = (self.name, self.subject, res) 
 115          return res

 116   


117 -    def evaluate(self, epoch):


118          return self.subject not in epoch.fast

 119   
 120   


121 -class leave_slow(common.predicate):


122      name = 'leave_slow' 
 123   


124 -    def precondition(self, epochs):


125          res = self.subject in epochs[0].slow 
 126          self.record = (self.name, self.subject, res) 
 127          return res

 128   


129 -    def evaluate(self, epoch):


130          return self.subject not in epoch.slow

 131   
 132   


133 -class is_active(common.predicate):


134      name = 'is_active' 
 135   


136 -    def evaluate(self, epoch):


137          return self.subject in epoch.actives

 138   
 139   


140 -class is_inactive(common.predicate):


141      name = 'is_inactive' 
 142   


143 -    def evaluate(self, epoch):


144          return self.subject in epoch.inactives

 145   
 146   


147 -class is_modulatory(common.predicate):


148      name = 'is_modulatory' 
 149   


150 -    def evaluate(self, epoch):


151          return self.subject in epoch.modulatory

 152   
 153   


154 -class is_most_dominant(common.predicate):


155      name = 'is_most_dominant' 
 156   


157 -    def evaluate(self, epoch):


158          return self.subject == epoch.actives[0]

 159   
 160   


161 -class is_fast(common.predicate):


162      name = 'is_fast' 
 163   


164 -    def evaluate(self, epoch):


165          return self.subject in epoch.fast

 166   
 167   


168 -class is_slow(common.predicate):


169      name = 'is_slow' 
 170   


171 -    def evaluate(self, epoch):


172          return self.subject in epoch.slow

 173   
 174   


175 -class is_order1(common.predicate):


176      name = 'is_order1' 
 177   


178 -    def evaluate(self, epoch):


179          return self.subject in epoch.order1

 180   
 181   
 182  # ------------------------------------------------- 
 183   
 184   


185 -def find_ep_ix(eps, t):


186      """Finds epoch during time t""" 
 187      for i, ep in enumerate(eps): 
 188          if t >= ep.t0: 
 189              try: 
 190                  test = t < eps[i+1].t0 
 191              except IndexError: 
 192                  test = t <= eps[i].t1 
 193              if test: 
 194                  return i 
 195          else: 
 196              raise ValueError("t must be in the epochs' time intervals")

 197   
 198   


199 -class regime(object):


200 -    def __init__(self, epochs, global_criteria, transition_criteria):


201          self.epochs = epochs 
 202          # all actives assumed to be the same 
 203          self.actives = epochs[0].actives 
 204          self.t0 = epochs[0].t0 
 205          self.t1 = epochs[-1].t1 
 206          # criteria are simple predicates or compound predicates built 
 207          # using and_op, or_op, not_op operator constructs 
 208          self.global_criteria = global_criteria 
 209          self.transition_criteria = transition_criteria

 210   
 211   


212 -def find_regime_transition(criteria_list, min_t=-np.Inf):


213      global_intervals = [] 
 214      transition_intervals = [] 
 215      record = {} 
 216      for criterion_ix, (epochs, global_criteria, transition_criteria) in enumerate(criteria_list): 
 217          check_global = global_criteria is not None 
 218          check_trans = transition_criteria is not None 
 219          # global criteria: record OK interval 
 220          # transition criteria: record last available ep.t1 in which post- 
 221          #  transition_criteria continue to hold using transition_criteria(ep) 
 222          #  and find earliest available overlap between all of these intervals 
 223          glt0 = np.Inf 
 224          glt1 = np.Inf 
 225          trt0 = np.Inf 
 226          trt1 = np.Inf 
 227          trans_found = False 
 228          #print "***" 
 229          if not check_global and not check_trans: 
 230              # nothing to check for this criterion! 
 231              continue 
 232          if check_trans: 
 233              if not transition_criteria.precondition(epochs): 
 234                  record[np.Inf] = (criterion_ix, None, 'transitional_precondition', (None, None), transition_criteria.record) 
 235                  return np.Inf, record 
 236          for epix, ep in enumerate(epochs): 
 237              if check_global and not global_criteria(ep) and ep.t1 >= min_t: 
 238                  if ep.t0 < min_t: 
 239                      # failed on first epoch 
 240                      record[np.Inf] = (criterion_ix, epix, 'global', (None, None), global_criteria.record) 
 241                      return np.Inf, record 
 242                  else: 
 243                      glt0 = min_t 
 244                      glt1 = ep.t0 
 245                      record[glt0] = (criterion_ix, epix, 'global', (glt0, glt1), global_criteria.record) 
 246                      #print "B", glt0, glt1 
 247                      break 
 248              if check_trans: 
 249                  if transition_criteria(ep): 
 250                      if not trans_found: 
 251                          if ep.t0 < min_t: 
 252                              if ep.t1 > min_t: 
 253                                  trans_found = True 
 254                                  trt0 = min_t 
 255                                  trt1 = ep.t1 
 256                          else: 
 257                              trt0 = ep.t0 
 258                              trt1 = ep.t1 
 259                              trans_found = True 
 260                      else: 
 261                          trt0 = max([min([trt0, ep.t0]), min_t]) 
 262                      trt1 = max([ep.t1, min_t]) 
 263                      #print "C", trt0, trt1 
 264                  elif trans_found: 
 265                      # may never be reached if end of epochs reached while 
 266                      # transition_criteria(ep) holds 
 267                      break 
 268          if trans_found: 
 269              record[trt0] = (criterion_ix, epix, 'transitional', (trt0, trt1), transition_criteria.record) 
 270          if check_global and (np.isfinite(glt0) or np.isfinite(glt1)): 
 271              global_intervals.append(Interval('glob', float, [glt0, glt1])) 
 272          if check_trans and (np.isfinite(trt0) or np.isfinite(trt1)): 
 273              transition_intervals.append(Interval('tran', float, [trt0, trt1])) 
 274      tri0 = Interval('tran_result', float, [-np.Inf, np.Inf]) 
 275      for i in transition_intervals: 
 276          #print "Trans", i[0], i[1] 
 277          try: 
 278              tri0 = tri0.intersect(i) 
 279          except: 
 280              if tri0 is None: 
 281                  print "No intersection for transition intervals" 
 282                  return np.Inf, record 
 283              else: 
 284                  raise 
 285      if tri0 is None: 
 286          print "No intersection for transition intervals" 
 287          return np.Inf, record 
 288      gli0 = Interval('glob_result', float, [-np.Inf, np.Inf]) 
 289      for i in global_intervals: 
 290          #print "Glob", i[0], i[1] 
 291          try: 
 292              gli0 = gli0.intersect(i) 
 293          except: 
 294              if gli0 is None: 
 295                  print "No intersection for global intervals" 
 296                  return np.Inf, record 
 297              else: 
 298                  raise 
 299      if gli0 is None: 
 300          print "No intersection for global intervals" 
 301          return np.Inf, record 
 302      any_trans = len(transition_intervals) > 0 
 303      any_global = len(global_intervals) > 0 
 304      if any_trans: 
 305          if any_global: 
 306              if gli0[1] >= tri0[0]: 
 307                  return tri0[0], record 
 308              else: 
 309                  return gli0[1], record 
 310          else: 
 311              return tri0[0], record 
 312      else: 
 313          if any_global: 
 314              return gli0[1], record 
 315          else: 
 316              return np.Inf, record

 317   
 318   
 319   
 320   


321 -class epoch(object):


322 -    def __init__(self, focus_var, sigma, gamma, actives, modulatory, inactives, 
 323                   order1, slow, fast, ever_slow, ever_fast, ranks, index0, index1, 
 324                   influences, rel_taus, traj_pts, inv_psi_namemap, inv_tau_namemap, 
 325                   relative_ratios=False, opts=None):


326          # defined by a seq of active variables (a domscales object) for a given 
 327          # focused variable, according to some influence threshold sigma. 
 328          # original influence values are influences pointset. 
 329          self.focus_var = focus_var 
 330          self.actives = actives 
 331          #self.actives.sort() 
 332          self.modulatory = modulatory 
 333          #self.modulatory.sort() 
 334          self.inactives = inactives 
 335          self.inactives.sort() 
 336          self.ranks = ranks 
 337          self.order1 = order1 
 338          self.slow = slow 
 339          self.fast = fast 
 340          self.ever_slow = ever_slow 
 341          self.ever_fast = ever_fast 
 342          self.traj_pts = traj_pts 
 343          self.sigma = sigma 
 344          self.gamma = gamma 
 345          # these maps take active names and restore prefixes to use in 
 346          # referencing Generator-created trajectory auxiliary variables 
 347          self.inv_psi_namemap = inv_psi_namemap 
 348          self.inv_tau_namemap = inv_tau_namemap 
 349          self.relative_ratios = relative_ratios 
 350          self.length = len(influences) 
 351          self.index0 = index0 
 352          self.index1 = index1 
 353          assert self.length == index1-index0+1 
 354          self.opts = opts 
 355          if traj_pts is not None: 
 356              self.t0 = traj_pts.indepvararray[0] 
 357              self.t1 = traj_pts.indepvararray[-1] 
 358          else: 
 359              self.t0 = None 
 360              self.t1 = None 
 361          self.influences = influences 
 362          # timescales relative to focus variable 
 363          self.rel_taus = rel_taus 
 364          self.index_interval = Interval('indices', int, [index0, index1]) 
 365          self.time_interval = Interval('time', float, [self.t0, self.t1])

 366   
 367   


368 -    def __cmp__(self, other):


369          if isinstance(other, epoch): 
 370              if self.focus_var == other.focus_var and self.sigma == other.sigma: 
 371                  return self.actives == other.actives and \ 
 372                         self.modulatory == other.modulatory and \ 
 373                         self.order1 == other.order1 and \ 
 374                         self.fast == other.fast and \ 
 375                         self.slow == other.slow 
 376          else: 
 377              raise TypeError("Only compare epochs to other epochs")

 378   
 379      __eq__ = __cmp__ 
 380   


381 -    def seq_str(self):


382          actives_w_speed = [] 
 383          for a in self.actives: 
 384              if a in self.fast: 
 385                  actives_w_speed.append(a+'[F]') 
 386              elif a in self.slow: 
 387                  actives_w_speed.append(a+'[S]') 
 388              else: 
 389                  actives_w_speed.append(a) 
 390          mod_w_speed = [] 
 391          for m in self.modulatory: 
 392              if m in self.fast: 
 393                  mod_w_speed.append(m+'[F]') 
 394              elif m in self.slow: 
 395                  mod_w_speed.append(m+'[S]') 
 396              else: 
 397                  mod_w_speed.append(m) 
 398          return actives_w_speed, mod_w_speed

 399   


400 -    def _infostr(self, verboselevel=0):


401          if self.opts.use_order: 
 402              uo_str = "(ord.)" 
 403          else: 
 404              uo_str = "(~ord.)" 
 405          str1 = "Epoch for %s at sigma %.5f %s: [%.5f, %.5f]" % \ 
 406               (self.focus_var, self.sigma, uo_str, self.t0, self.t1) 
 407          if verboselevel == 0: 
 408              return str1 
 409          else: 
 410              actives_w_speed, mod_w_speed = self.seq_str() 
 411              str2 = " Actives: " + ",".join(actives_w_speed) 
 412              str3 = "   Modulatory: " + ",".join(mod_w_speed) 
 413              return str1 + str2 + str3

 414   


415 -    def info(self, verboselevel=1):


416          print self._infostr(verboselevel)

 417   


418 -    def __str__(self):


419          return self._infostr(0)

 420   
 421      __repr__ = __str__

 422   
 423   


424 -def _comp(a, b, eps, atol):


425      try: 
 426          rat = a/b 
 427      except ZeroDivisionError: 
 428          rat = np.Inf 
 429      return np.allclose(rat, eps, atol=atol)

 430   
 431   


432 -def transition_psi(epoch, pt, atol):


433      """Assume that, generically, there will only be one transition at a time. 
 434      """ 
 435      acts = pt[epoch.inv_psi_namemap(epoch.actives)] 
 436      # ensures order is the same 
 437      acts_plain = epoch.inv_psi_namemap.inverse()(acts.coordnames) 
 438      if epoch.modulatory == []: 
 439          mods = [] 
 440          mods_plain = [] 
 441      else: 
 442          mods = pt[epoch.inv_psi_namemap(epoch.modulatory)] 
 443          mods_plain = epoch.inv_psi_namemap.inverse()(mods.coordnames) 
 444      relrat = epoch.relative_ratios 
 445      leavers = [] 
 446      joiners = [] 
 447      if relrat: 
 448          # leavers 
 449          if len(acts) > 1: 
 450              ptixs = np.argsort(acts)[::-1] 
 451              for i in range(len(ptixs)-1): 
 452                  if _comp(acts[ptixs[i]], acts[ptixs[i+1]], epoch.sigma, atol): 
 453                      leavers.append(acts_plain[i+1]) 
 454          # else: can't be any leavers (case caught by joiners) 
 455          # joiners 
 456          min_act = min(acts) 
 457          for i, m in enumerate(mods): 
 458              if _comp(min_act, m, epoch.sigma, atol): 
 459                  joiners.append(mods_plain[i]) 
 460      else: 
 461          max_ix = np.argmax(acts) 
 462          min_ix = np.argmin(acts) 
 463          # leavers 
 464          if _comp(acts[max_ix], acts[min_ix], epoch.sigma, atol): 
 465              leavers.append(acts_plain[min_ix]) 
 466          # joiners 
 467          for i, m in enumerate(mods): 
 468              if _comp(acts[max_ix], m, epoch.sigma, atol): 
 469                  joiners.append(mods_plain[i]) 
 470      if len(leavers) + len(joiners) == 0: 
 471          raise PyDSTool_ValueError("No transition found: Tolerance too small?") 
 472      elif len(leavers) + len(joiners) > 1: 
 473          raise PyDSTool_ValueError("Too many transitions found: Tolerance too large?") 
 474      else: 
 475          if len(leavers) == 1: 
 476              return ('leave', leavers[0]) 
 477          else: 
 478              return ('join', joiners[0])

 479   
 480   


481 -def transition_tau(epoch, pt, atol):


482      """Assume that, generically, there will only be one transition at a time. 
 483      """ 
 484      if epoch.slow == []: 
 485          slow = [] 
 486          slow_plain = [] 
 487      else: 
 488          slow = pt[epoch.inv_tau_namemap(epoch.slow)] 
 489          slow_plain = epoch.inv_tau_namemap.inverse()(slow.coordnames) 
 490      if epoch.fast == []: 
 491          fast = [] 
 492          fast_plain = [] 
 493      else: 
 494          fast = pt[epoch.inv_tau_namemap(epoch.fast)] 
 495          fast_plain = epoch.inv_tau_namemap.inverse()(fast.coordnames) 
 496      if epoch.order1 == []: 
 497          order1 = [] 
 498          order1_plain = [] 
 499      else: 
 500          order1 = pt[epoch.inv_tau_namemap(epoch.order1)] 
 501          order1_plain = epoch.inv_tau_namemap.inverse()(order1.coordnames) 
 502      slow_leavers = [] 
 503      slow_joiners = [] 
 504      max_ix = np.argmax(slow) 
 505      min_ix = np.argmin(slow) 
 506      # slow leavers -> order1 
 507      if _comp(slow[min_ix], 1, epoch.gamma, atol): 
 508          slow_leavers.append(slow_plain[min_ix]) 
 509      # slow joiners <- order1 
 510      for i, m in enumerate(order1): 
 511          if _comp(m, 1, epoch.gamma, atol): 
 512              slow_joiners.append(order1_plain[i]) 
 513      # 
 514      max_ix = np.argmax(fast) 
 515      min_ix = np.argmin(fast) 
 516      # fast leavers -> order1 
 517      fast_leavers = [] 
 518      fast_joiners = [] 
 519      if _comp(1, fast[min_ix], epoch.gamma, atol): 
 520          fast_leavers.append(fast_plain[min_ix]) 
 521      # fast joiners <- order1 
 522      for i, m in enumerate(order1): 
 523          if _comp(1, m, epoch.gamma, atol): 
 524              fast_joiners.append(order1_plain[i]) 
 525      len_slow_l = len(slow_leavers) 
 526      len_slow_j = len(slow_joiners) 
 527      len_fast_l = len(fast_leavers) 
 528      len_fast_j = len(fast_joiners) 
 529      if len_slow_l + len_slow_j + \ 
 530         len_fast_l + len_fast_j == 0: 
 531          raise PyDSTool_ValueError("No transition found: Tolerance too small?") 
 532      elif len_slow_l + len_slow_j + \ 
 533           len_fast_l + len_fast_j > 1: 
 534          raise PyDSTool_ValueError("Too many transitions found: Tolerance too large?") 
 535      else: 
 536          if len_slow_l == 1: 
 537              return ('slow_leave', slow_leavers[0]) 
 538          elif len_slow_j == 1: 
 539              return ('slow_join', slow_joiners[0]) 
 540          elif len_fast_l == 1: 
 541              return ('fast_leave', fast_leavers[0]) 
 542          else: 
 543              return ('fast_join', fast_joiners[0])

 544   


545 -def check_opts(opts):


546      def_vals = {'use_order': True, 'speed_fussy': True} 
 547      ok_keys = def_vals.keys() 
 548      if opts is None: 
 549          return common.args(**def_vals) 
 550      else: 
 551          for k in ok_keys: 
 552              if k not in opts.keys(): 
 553                  opts[k] = def_vals[k] 
 554          rem_keys = common.remain(opts.keys(), ok_keys) 
 555          if rem_keys != []: 
 556              raise ValueError("Invalid options passed in opts argument: %s" % rem_keys) 
 557          else: 
 558              return opts

 559   
 560   


561 -class domscales(object):


562 -    def __init__(self, focus_var, traj_pts, influence_pts, 
 563                   influence_type, tau_refs, psi_refs, opts=None):


564          ## !!! Assumes Psi-type influences only 
 565          self.focus_var = focus_var 
 566          self.traj_pts = traj_pts 
 567          self.tau_refs = tau_refs 
 568          base = 'tau_' 
 569          avoid_prefix = len(base) 
 570          tau_namedict = {} 
 571          for c in traj_pts.coordnames: 
 572              if c[:avoid_prefix] == base: 
 573                  tau_namedict[c] = c[avoid_prefix:] 
 574          self.tau_namemap = parseUtils.symbolMapClass(tau_namedict) 
 575          self.psi_refs = psi_refs 
 576          base = 'psi_'+focus_var+'_' 
 577          avoid_prefix = len(base) 
 578          psi_namedict = {} 
 579          for c in influence_pts.coordnames: 
 580              psi_namedict[c] = c[avoid_prefix:] 
 581          self.psi_namemap = parseUtils.symbolMapClass(psi_namedict) 
 582          influence_pts.mapNames(self.psi_namemap) 
 583          self.influence_pts = influence_pts 
 584          # rank largest first 
 585          ranks = np.argsort(influence_pts.coordarray.T)[:,::-1] 
 586          greatest_vals = np.array([p[ranks[i,0]] for i, p in \ 
 587                                    enumerate(influence_pts.coordarray.T)]) 
 588          self.ranks = ranks 
 589          self.greatest_vals = greatest_vals 
 590          # normalized at each time point (largest = 1) 
 591          self.normed_coordarray = influence_pts.coordarray/greatest_vals 
 592          self.influence_type = influence_type 
 593          self.coordnames = influence_pts.coordnames 
 594          self.num_pts = len(influence_pts) 
 595          self.opts = check_opts(opts)

 596   
 597   


598 -    def calc_epochs(self, sigma, gamma, relative_ratios=False):


599          """Calculate epochs without cycle checking""" 
 600          assert sigma > 1, "sigma parameter must be > 1" 
 601          assert gamma > 1, "gamma parameter must be > 1" 
 602          epochs = [] 
 603          old_actives = [] 
 604          old_fast = [] 
 605          old_slow = [] 
 606          # we just use this dictionary to store unique mod names 
 607          # the values are irrelevant 
 608          all_modulatory = {} 
 609          ep_start = 0 
 610          complete_epoch = False 
 611          for i in xrange(self.num_pts): 
 612              # ignore_change is used to avoid single time-point epochs: 
 613              # it ensures 'old' actives and time scale sets don't get updated for 
 614              # the single time-point change 
 615              ignore_change = False 
 616              ds_pt = self.get_domscales_point(i, relative_ratios) 
 617              groups = split(ds_pt, sigma) 
 618              actives = groups[0].coordnames 
 619              if not self.opts.use_order: 
 620                  actives.sort() 
 621              # gather all modulatory variables 
 622              if len(groups) > 1: 
 623                  modulatory = groups[1].coordnames 
 624                  if not self.opts.use_order: 
 625                      modulatory.sort() 
 626              else: 
 627                  modulatory = [] 
 628              if self.opts.speed_fussy: 
 629                  tau = get_taus(self.traj_pts[i], self.tau_refs) 
 630                  tau_ref = tau[self.focus_var] 
 631                  rel_tau = tau/tau_ref 
 632                  slow_test = rel_tau/gamma > 1 
 633                  fast_test = rel_tau*gamma < 1 
 634                  cs = tau.coordnames 
 635                  slow = common.intersect([cs[ci] for ci in range(len(cs)) if slow_test[ci]], actives) 
 636                  fast = common.intersect([cs[ci] for ci in range(len(cs)) if fast_test[ci]], actives) 
 637                  if i == 0: 
 638                      if self.num_pts == 1: 
 639                          complete_epoch = True 
 640                          act_vars = actives 
 641                          mod_vars = modulatory 
 642                          i_stop = 1 
 643                  elif not (len(old_fast) == len(fast) and common.remain(old_fast, fast) == [] and \ 
 644                            len(old_slow) == len(slow) and common.remain(old_slow, slow) == []): 
 645                      # non-identical sets 
 646                      if i - ep_start > 1: 
 647                          complete_epoch = True 
 648                      else: 
 649                          # ignore epoch made up of single time point 
 650                          ignore_change = True 
 651                      act_vars = old_actives 
 652                      mod_vars = all_modulatory.keys() 
 653                      i_stop = i 
 654                  if ignore_change: 
 655                      old_fast = fast 
 656                      old_slow = slow 
 657                  else: 
 658                      old_fast = common.makeSeqUnique(utils.union(old_fast,fast)) 
 659                      old_slow = common.makeSeqUnique(utils.union(old_slow,slow)) 
 660              if old_actives == actives and i < self.num_pts-1: 
 661                  all_modulatory.update(dict(zip(modulatory,enumerate(modulatory)))) 
 662              else: 
 663                  # epoch changed or we reached the last point 
 664                  if old_actives == [] and self.num_pts > 1: 
 665                      # first epoch: don't make an epoch object until it ends 
 666                      all_modulatory.update(dict(zip(modulatory,enumerate(modulatory)))) 
 667                  elif i - ep_start == 1 and i < self.num_pts-1: 
 668                      # ignore epoch made up of single time point 
 669                      all_modulatory.update(dict(zip(modulatory,enumerate(modulatory)))) 
 670                      ignore_change = True 
 671                  else: 
 672                      if self.num_pts == 1: 
 673                          act_vars = actives 
 674                          mod_vars = modulatory 
 675                          i_stop = 1 
 676                      else: 
 677                          act_vars = old_actives 
 678                          mod_vars = all_modulatory.keys() 
 679                          i_stop = i 
 680                      complete_epoch = True 
 681                  if ignore_change: 
 682                      old_actives = common.makeSeqUnique(utils.union(old_actives, actives)) 
 683                  else: 
 684                      # update old_actives now that it has changes 
 685                      old_actives = actives 
 686              if complete_epoch: 
 687                  # reset 
 688                  complete_epoch = False 
 689                  influences = self.influence_pts[ep_start:i_stop] 
 690                  avg_influence = {} 
 691                  for a in act_vars: 
 692                      avg_influence[a] = np.mean(influences[a]) 
 693                  # get approximate order 
 694                  act_vars = common.sortedDictLists(avg_influence, reverse=True)[0] 
 695                  avg_influence = {} 
 696                  not_mod = [] 
 697                  for m in mod_vars: 
 698                      avg = np.mean(influences[m]) 
 699                      if avg > 0: 
 700                          avg_influence[m] = avg 
 701                      else: 
 702                          # influence actually zero so actually should be in 
 703                          # inactive set 
 704                          not_mod.append(m) 
 705                  mod_vars = common.remain(mod_vars, not_mod) 
 706                  # get approximate order 
 707                  mod_vars = common.sortedDictLists(avg_influence, 
 708                                                    reverse=True)[0] 
 709                  inact_vars = common.remain(self.coordnames, 
 710                                             act_vars+mod_vars) 
 711                  pts = self.traj_pts[ep_start:i_stop] 
 712                  order1, slow, fast, ever_slow, ever_fast, rel_taus = \ 
 713                        self.calc_fast_slow(get_taus(pts, self.tau_refs), 
 714                                            gamma) 
 715                  rel_taus.mapNames(self.tau_namemap) 
 716                  epochs.append(epoch(self.focus_var, sigma, gamma, 
 717                                      act_vars, mod_vars, inact_vars, 
 718                                      order1, slow, fast, ever_slow, ever_fast, 
 719                                      self.ranks[ep_start:i_stop,:], 
 720                                      ep_start, i_stop-1, 
 721                                      self.influence_pts[ep_start:i_stop], rel_taus, 
 722                                      pts, relative_ratios=ds_pt.relative_ratios, 
 723                                      inv_psi_namemap=self.psi_namemap.inverse(), 
 724                                      inv_tau_namemap=self.tau_namemap.inverse(), 
 725                                      opts=self.opts)) 
 726                  ep_start = i  # doesn't need to be updated for num_pts == 1 
 727                  all_modulatory = {} 
 728          self.epochs = epochs

 729   
 730   


731 -    def calc_fast_slow(self, taus, gamma):


732          """Determines variables which are always fast and slow, and those 
 733          which are ever fast and slow, over a given set of time scale points. 
 734          """ 
 735          focus_taus = taus[self.focus_var] 
 736          rel_taus = taus/focus_taus 
 737          cs = taus.coordnames 
 738          ixs = xrange(len(cs)) 
 739          n = xrange(len(taus)) 
 740          # these tests can never select focus_var which has 
 741          # been normalized to tau = 1 
 742          slow_test = rel_taus/gamma > 1 
 743          fast_test = rel_taus*gamma < 1 
 744          slow_array = [[i for i in ixs if slow_test[i,j]] for j in n] 
 745          fast_array = [[i for i in ixs if fast_test[i,j]] for j in n] 
 746          slow_ixs = ever_slow_ixs = slow_array[0] 
 747          for row in slow_array[1:]: 
 748              slow_ixs = utils.intersect(slow_ixs, row) 
 749              ever_slow_ixs = np.unique(utils.union(ever_slow_ixs, row)) 
 750          slow_vars = [cs[i] for i in slow_ixs] 
 751          ever_slow_vars = [cs[i] for i in ever_slow_ixs] 
 752          fast_ixs = ever_fast_ixs = fast_array[0] 
 753          for row in fast_array[1:]: 
 754              fast_ixs = utils.intersect(fast_ixs, row) 
 755              ever_fast_ixs = np.unique(utils.union(ever_fast_ixs, row)) 
 756          fast_vars = [cs[i] for i in fast_ixs] 
 757          ever_fast_vars = [cs[i] for i in ever_fast_ixs] 
 758          order1_vars = utils.remain(cs, slow_vars+fast_vars) 
 759          return order1_vars, slow_vars, fast_vars, \ 
 760                 list(ever_slow_vars), list(ever_fast_vars), rel_taus

 761   
 762   


763 -    def lookup_index(self, ix):


764          """Locates index ix in the epochs, returning the epoch index, 
 765          epoch object, and the index within the epoch's pointset data.""" 
 766          assert self.epochs 
 767          for epix, ep in enumerate(self.epochs): 
 768              if ix >= ep.index0 and ix <= ep.index1: 
 769                  return epix, ep, ix - ep.index0

 770   


771 -    def lookup_time(self, t):


772          """Locates time t in the epochs, returning the epoch index, 
 773          epoch object, and the approximate corresponding index within 
 774          the epoch's pointset data. 
 775          Note, because of the time discretization, time is compared up to 
 776          the next epoch's start time.""" 
 777          assert self.epochs 
 778          for epix, ep in enumerate(self.epochs): 
 779              try: 
 780                  next_time = self.epochs[epix+1].t0 
 781                  comp = operator.lt 
 782              except IndexError: 
 783                  next_time = ep.t1 
 784                  comp = operator.le 
 785              if t >= ep.t0 and comp(t, next_time): 
 786                  ix = ep.influences.find(t, end=0) 
 787                  return epix, ep, ix

 788   
 789   


790 -    def calc_regimes(self, options=None):


791          assert self.epochs 
 792          raise NotImplementedError

 793   
 794   


795 -    def get_domscales_point(self, i, relative_ratios):


796          ds_pt = common.args(relative_ratios=relative_ratios) 
 797          ds_pt.coordnames = list(np.array(self.coordnames)[self.ranks[i]]) 
 798          # influences are in rank order (largest first) 
 799          ds_pt.influences = list(self.influence_pts.coordarray[self.ranks[i],i]) 
 800          # diameter measures ratio of largest to smallest influence 
 801          try: 
 802              ds_pt.diameter = ds_pt.influences[0]/ds_pt.influences[-1] 
 803          except ZeroDivisionError: 
 804              ds_pt.diameter = np.inf 
 805          ds_pt.relative_influences = list(self.normed_coordarray[self.ranks[i],i]) 
 806          if relative_ratios: 
 807              as_ratios = as_relative_ratios 
 808          else: 
 809              as_ratios = as_absolute_ratios 
 810          ds_pt.as_ratios = as_ratios(ds_pt.influences) 
 811          return ds_pt

 812   
 813   


814 -def define_psi_events(acts_all, mods_all, focus_var, ignore_transitions=None):


815      if ignore_transitions is None: 
 816          acts_leave = acts_all 
 817          mods_join = mods_all 
 818      else: 
 819          remove_acts = [] 
 820          remove_mods = [] 
 821          for trans, var in ignore_transitions: 
 822              if trans == 'leave': 
 823                  remove_acts.append(var) 
 824              elif trans == 'join': 
 825                  remove_mods.append(var) 
 826              else: 
 827                  raise ValueError("Invalid transition type: %s" % trans) 
 828          acts_leave = [a for a in acts_all if a not in remove_acts] 
 829          mods_join = [m for m in mods_all if m not in remove_mods] 
 830      act_coordlist = ['psi_'+focus_var+'_'+c for c in acts_all] 
 831      act_leave_coordlist = ['psi_'+focus_var+'_'+c for c in acts_leave] 
 832      mod_coordlist = ['psi_'+focus_var+'_'+c for c in mods_join] 
 833      evdefs = {} 
 834      if len(act_leave_coordlist) > 0 and len(act_coordlist) > 0: 
 835          if len(act_coordlist) == 1: 
 836              term1 = '(' + str(act_coordlist[0]) + ')' 
 837          else: 
 838              term1 = "max( [ %s ] )" % ",".join(act_coordlist) 
 839          if len(act_leave_coordlist) == 1: 
 840              term2 = '(' + str(act_leave_coordlist[0]) + ')' 
 841          else: 
 842              term2 = "min( [ %s ] )" % ",".join(act_leave_coordlist) 
 843          act_leave_def = "%s / %s - dssrt_sigma" % (term1, term2) 
 844          evdefs['act_leave_ev'] = common.args(defn=act_leave_def, dirn=1, pars=['dssrt_sigma']) 
 845      if len(act_coordlist) > 0 and len(mod_coordlist) > 0: 
 846          if len(act_coordlist) == 1: 
 847              term1 = '(' + str(act_coordlist[0]) + ')' 
 848          else: 
 849              term1 = "max( [ %s ] )" % ",".join(act_coordlist) 
 850          if len(mod_coordlist) == 1: 
 851              term2 = '(' + str(mod_coordlist[0]) + ')' 
 852          else: 
 853              term2 = "max( [ %s ] )" % ",".join(mod_coordlist) 
 854          mod_join_def = "%s / %s - dssrt_sigma" % (term1, term2) 
 855          evdefs['mod_join_ev'] = common.args(defn=mod_join_def, dirn=-1, pars=['dssrt_sigma']) 
 856      return evdefs

 857   
 858   


859 -def define_tau_events(slow, fast, order1, ref):


860      ref_name = 'tau_'+ref 
 861      assert ref in order1, "reference time scale must be in order1 list" 
 862      evdefs = {} 
 863      if slow != []: 
 864          slow_list = ", ".join(['tau_'+c for c in slow]) 
 865          if len(slow) == 1: 
 866              slow_leave_def = "(%s)/(%s) - dssrt_gamma" % (slow_list[0], ref_name) 
 867          else: 
 868              slow_leave_def = "min([ %s ])/(%s) - dssrt_gamma" % (slow_list, ref_name) 
 869          evdefs['slow_leave_ev'] = common.args(defn=slow_leave_def, dirn=-1, pars=['dssrt_gamma']) 
 870      if fast != []: 
 871          fast_list = ", ".join(['tau_'+c for c in fast]) 
 872          if len(fast) == 1: 
 873              fast_leave_def = "(%s)/(%s) - 1./dssrt_gamma" % (fast_list[0], ref_name) 
 874          else: 
 875              fast_leave_def = "max([ %s ])/(%s) - 1./dssrt_gamma" % (fast_list, ref_name) 
 876          evdefs['fast_leave_ev'] = common.args(defn=fast_leave_def, dirn=1, pars=['dssrt_gamma']) 
 877      other_o1 = common.remain(order1, ref) 
 878      if other_o1 != []: 
 879          o1_list = ", ".join(['tau_'+c for c in other_o1]) 
 880          if len(other_o1) == 1: 
 881              slow_join_def = "(%s)/(%s) - dssrt_gamma" % (o1_list[0], ref_name) 
 882              fast_join_def = "(%s)/(%s) - 1./dssrt_gamma" % (o1_list[0], ref_name) 
 883          else: 
 884              slow_join_def = "max([ %s ])/(%s) - dssrt_gamma" % (o1_list, ref_name) 
 885              fast_join_def = "min([ %s ])/(%s) - 1./dssrt_gamma" % (o1_list, ref_name) 
 886          evdefs['slow_join_ev'] = common.args(defn=slow_join_def, dirn=1, pars=['dssrt_gamma']) 
 887          evdefs['fast_join_ev'] = common.args(defn=fast_join_def, dirn=-1, pars=['dssrt_gamma']) 
 888      return evdefs

 889   
 890   


891 -class dssrt_assistant(object):


892 -    def __init__(self, kw):


893          model = kw['model'] 
 894          if isinstance(model, HybridModel): 
 895              # check that there is a single sub-model (not a true hybrid system) 
 896              assert len(model.registry) == 1 
 897              self.model = model.registry.values()[0] 
 898              self.gen = self.model.registry.values()[0] 
 899          elif isinstance(model, NonHybridModel): 
 900              assert len(model.registry) == 1 
 901              self.model = model 
 902              self.gen = self.model.registry.values()[0] 
 903          else: 
 904              # assume generator 
 905              self.model = None 
 906              self.gen = model 
 907          FScompatMap = self.gen._FScompatibleNames 
 908          self.reset() 
 909          self.gamma1 = {} 
 910          self.gamma2 = {} 
 911          self.pars = self.gen.pars #FScompatMap(self.gen.pars) 
 912          if self.model is None or self.model._mspecdict is None: 
 913              # dict of each variable's inputs (FScompatMap will copy) 
 914              inputs = kw['inputs'] #FScompatMap(kw['inputs']) 
 915              # dummy input names such as Lk for leak channel, which 
 916              # has no activation variable, will not be present in 
 917              # the FScompatMap, so use this opportunity to update 
 918              # the mapping 
 919              for k, gam_inps in inputs.items(): 
 920                  gamma1 = [] 
 921                  try: 
 922                      for i in gam_inps.gamma1: 
 923                          if '.' in i and i not in FScompatMap: 
 924                              i_FSc = i.replace('.','_') 
 925                              FScompatMap[i] = i_FSc 
 926                          #    gamma1.append(i_FSc) 
 927                          #else: 
 928                          gamma1.append(i) 
 929                  except AttributeError: 
 930                      pass 
 931                  self.gamma1[k] = gamma1 
 932                  gamma2 = [] 
 933                  try: 
 934                      for i in gam_inps.gamma2: 
 935                          if '.' in i and i not in FScompatMap: 
 936                              i_FSc = i.replace('.','_') 
 937                              FScompatMap[i] = i_FSc 
 938                          #    gamma2.append(i_FSc) 
 939                          #else: 
 940                          gamma2.append(i) 
 941                  except AttributeError: 
 942                      pass 
 943                  self.gamma2[k] = gamma2 
 944              # all_vars includes 'mock' variables 
 945              # given by auxiliary functions 
 946              self.all_vars = inputs.keys() 
 947          else: 
 948              # deduce them from the dependencies in the 
 949              # ModelSpec dict 
 950              #self.inputs = ? 
 951              # acquire auxiliary fn names 
 952              #self.aux_fns = ? 
 953              # acquire types of inputs (gamma sets) 
 954              #self.gamma1 = ? 
 955              #self.gamma2 = ? 
 956              # Can expect only mspec 
 957              self._init_from_MSpec(self.model._mspecdict.values()[0].modelspec) 
 958          all_inputs = [] 
 959          for ins in self.gamma1.values() + self.gamma2.values(): 
 960              try: 
 961                  all_inputs.extend(ins) 
 962              except TypeError: 
 963                  # ins is None 
 964                  pass 
 965          self._FScompatMap = FScompatMap 
 966          self._FScompatMapInv = FScompatMap.inverse() 
 967          self.all_inputs = np.unique(all_inputs).tolist() 
 968          self.tau_refs = kw['taus'] 
 969          self.inf_refs = kw['infs'] 
 970          self.psi_refs = kw['psis'] 
 971          opts = common.args() 
 972          try: 
 973              opts.use_order = kw['use_order'] 
 974          except KeyError: 
 975              pass 
 976          try: 
 977              opts.speed_fussy = kw['speed_fussy'] 
 978          except KeyError: 
 979              pass 
 980          self.opts = opts 
 981          self._setup_taus_psis()

 982   


983 -    def __getstate__(self):


984          d = copy.copy(self.__dict__) 
 985          del d['taus'] 
 986          del d['taus_direct'] 
 987          del d['infs'] 
 988          del d['infs_direct'] 
 989          del d['psis'] 
 990          del d['psis_direct'] 
 991          return d

 992   


993 -    def __setstate__(self):


994          self.__dict__.update(state) 
 995          self._setup_taus_psis()

 996   


997 -    def _setup_taus_psis(self):


998          # process these to extract whether these are parameters, expressions 
 999          # or references to auxiliary functions and convert them to callable 
1000          # objects 
1001          # 
1002          # also, every reference to an auxiliary variable must be mappable to 
1003          # a dssrt_fn_X aux function to allow direct calculating of a tau or 
1004          # Psi from only state variables in a Point. 
1005          # ... this needs a parallel set of definitions to be created 
1006          self._direct_fail = False 
1007          d = {} 
1008          fspecs = self.gen.funcspec._auxfnspecs 
1009          fn_base = 'dssrt_fn_' 
1010          for var, tau in self.tau_refs.items(): 
1011              if tau is None: 
1012                  continue 
1013              # signature for the aux fn is in the Generator's FuncSpec, e.g. 
1014              # 'Na_dssrt_fn_cond': (['m', 'h'], 'Na_g*m*m*m*h') 
1015              # 'Na_dssrt_fn_tauh': (['V'], '1/(0.128*exp(-(50.0+V)/18)+4/(1+exp(-(V+27.0)/5)))') 
1016              # for which prefix is 'Na' 
1017              v_hier_list = var.split('.') 
1018              prefix = '_'.join(v_hier_list[:-1])  # empty if no hierarchical name 
1019              var_postfix = v_hier_list[-1] 
1020              if len(prefix) > 0: 
1021                  fname = prefix+'_'+fn_base+'tau'+var_postfix 
1022              else: 
1023                  fname = fn_base+'tau'+var_postfix 
1024              # convert fn sig names to resolvable hierarchical names using knowledge 
1025              # of inputs 
1026              inps_to_var = self.gamma1[var] + self.gamma2[var] 
1027              try: 
1028                  sig = fspecs[fname][0] 
1029              except KeyError: 
1030                  # dssrt_ functions were not defined 
1031                  self._direct_fail = True 
1032                  break 
1033              # match sig entries to final elements of inps_to_var 
1034              for inp in inps_to_var: 
1035                  inp_hier_list = inp.split('.') 
1036                  try: 
1037                      ix = sig.index(inp_hier_list[-1]) 
1038                  except ValueError: 
1039                      # postfix not in sig 
1040                      pass 
1041                  else: 
1042                      sig[ix] = inp 
1043              d[tau.replace('.','_')] = fname+'(' + ",".join(sig) + ')' 
1044   
1045          for var, inf in self.inf_refs.items(): 
1046              if inf is None: 
1047                  continue 
1048              # signature for the aux fn is in the Generator's FuncSpec, e.g. 
1049              # 'Na_dssrt_fn_cond': (['m', 'h'], 'Na_g*m*m*m*h') 
1050              # 'Na_dssrt_fn_tauh': (['V'], '1/(0.128*exp(-(50.0+V)/18)+4/(1+exp(-(V+27.0)/5)))') 
1051              # for which prefix is 'Na' 
1052              v_hier_list = var.split('.') 
1053              prefix = '_'.join(v_hier_list[:-1])  # empty if no hierarchical name 
1054              var_postfix = v_hier_list[-1] 
1055              if len(prefix) > 0: 
1056                  fname = prefix+'_'+fn_base+var_postfix+'inf' 
1057              else: 
1058                  fname = fn_base+var_postfix+'inf' 
1059              # convert fn sig names to resolvable hierarchical names using knowledge 
1060              # of inputs 
1061              inps_to_var = self.gamma1[var] + self.gamma2[var] 
1062              try: 
1063                  sig = fspecs[fname][0] 
1064              except KeyError: 
1065                  # dssrt_ functions were not defined 
1066                  self._direct_fail = True 
1067                  break 
1068              # match sig entries to final elements of inps_to_var 
1069              for inp in inps_to_var: 
1070                  inp_hier_list = inp.split('.') 
1071                  try: 
1072                      ix = sig.index(inp_hier_list[-1]) 
1073                  except ValueError: 
1074                      # postfix not in sig 
1075                      pass 
1076                  else: 
1077                      sig[ix] = inp 
1078              d[inf.replace('.','_')] = fname+'(' + ",".join(sig) + ')' 
1079   
1080          self._direct_ref_mapping = parseUtils.symbolMapClass(d) 
1081          FScMap = self._FScompatMap 
1082          self.taus = {} 
1083          self.taus_direct = {} 
1084          for k, v in self.tau_refs.items(): 
1085              self.taus[k], self.taus_direct[k] = self._process_expr(FScMap(v)) 
1086   
1087          self.infs = {} 
1088          self.infs_direct = {} 
1089          for k, v in self.inf_refs.items(): 
1090              self.infs[k], self.infs_direct[k] = self._process_expr(FScMap(v)) 
1091   
1092          self.psis = {} 
1093          self.psis_direct = {} 
1094          for k, vdict in self.psi_refs.items(): 
1095              if vdict is None: 
1096                  self.psis[k], self.psis_direct[k] = (None, None) 
1097              else: 
1098                  self.psis[k] = {} 
1099                  self.psis_direct[k] = {} 
1100                  for inp, psiv in vdict.items(): 
1101                      self.psis[k][inp], self.psis_direct[k][inp] = \ 
1102                                              self._process_expr(FScMap(psiv))

1103   
1104   


1105 -    def _init_from_MSpec(self, mspec):


1106          ## !!! 
1107          # get Psi formulae by differentiating x_inf directly 
1108          #deriv_list = [Symbolic.Diff(,i) for i in all_var_inputs] 
1109          raise NotImplementedError

1110   
1111   


1112 -    def _process_expr(self, expr):


1113          """Create two Symbolic.Fun(ction) versions of the given expression: 
1114   
1115          (1) The first is intended for processing post-integration 
1116          pointsets to yield the dominant scale Psi quantities, 
1117          when the pointsets contain already-computed auxiliary variables 
1118          for taus, infs, etc. 
1119   
1120          (2) The second is intended for on-demand calculation of Psi's 
1121          when only the system's state variables are given, thus 
1122          taus and infs etc. must be computed this class. If user did not 
1123          provide the dssrt_fn functions this will be ignored and not created. 
1124   
1125          The returned pair of pairs is 
1126           ((arg_list_signature (1), symbolic function (1)), 
1127            (arg_list_signature (2), symbolic function (2))) 
1128          unless None was passed (in which case None is returned). 
1129          """ 
1130          if expr is None: 
1131              # None 
1132              return (None, None) 
1133          elif parseUtils.isNumericToken(expr): 
1134              # numeric literal 
1135              f = Symbolic.expr2fun(expr) 
1136              return ( ([], f), ([], f) ) 
1137          elif expr in self.pars: 
1138              # parameter 
1139              val = 'refpars["%s"]' % expr 
1140              defs = {'refpars': self.pars} 
1141              f = Symbolic.expr2fun(val, **defs) 
1142              return ( ([], f), ([], f) ) 
1143          elif expr in self.gen.inputs: 
1144              # input literal - turn into a function of t 
1145              f = Symbolic.expr2fun(expr+'(t)') 
1146              return ( (['t'], f), (['t'], f) ) 
1147          else: 
1148              # test for valid math expression 
1149              # - may include auxiliary function call (with arguments) 
1150              fnames = self.gen.auxfns.keys() 
1151              themap = dict(self.gen.auxfns.items()) 
1152              themap.update(dict(self.gen.inputs.items())) 
1153              defs = {'refpars': self.pars} 
1154              pnames = self.pars.keys() 
1155              pvals = ['refpars["%s"]' % pn for pn in pnames] 
1156  #            parmap = dict(zip(pnames, 
1157  #                              [Symbolic.expr2fun(v, **defs) for v in pvals])) 
1158              #val, dummies = parseUtils.replaceCallsWithDummies(expr, fnames) 
1159              val = expr   # retained in case revert to replaceCallsWithDummies(...) 
1160              #dummies = {} 
1161              val_q = Symbolic.QuantSpec('__expr__', val) 
1162              mapping = dict(zip(pnames, pvals)) 
1163              used_inputs = utils.intersect(val_q.parser.tokenized, 
1164                                 self.gen.inputs.keys()) 
1165              for inp in used_inputs: 
1166                  # turn into a function of t 
1167                  mapping[inp] = inp + '(t)' 
1168              val_q.mapNames(mapping) 
1169              #map.update(parmap) 
1170              # Don't update with self.pars otherwise param values are hard-wired 
1171  #            map.update(self.pars) 
1172  #            for num, dummy in dummies.items(): 
1173  #                dstr = '__dummy%i__' % num 
1174  #                dspec = Symbolic.QuantSpec('__temp__', dummy) 
1175  #                defs[dstr] = Symbolic.expr2fun(dspec, **map) 
1176              #defs.update(parmap) 
1177              #defs.update(self.pars) 
1178   
1179              # Every reference to an auxiliary variable must be mappable to 
1180              # a dssrt_fn_X aux function to allow direct calculating of a tau or 
1181              # Psi from only state variables in a Point. 
1182              if not self._direct_fail: 
1183                  val_q_direct = copy.copy(val_q) 
1184                  val_q_direct.mapNames(self._direct_ref_mapping) 
1185   
1186              defs.update(themap) 
1187              if val in defs: 
1188                  # then outermost level is just a single function call 
1189                  # which has been replaced unnecessarily by a dummy term 
1190                  fn = copy.copy(defs[val]) 
1191                  del defs[val] 
1192                  return ( (fn._args, fn), (fn._args, fn) ) 
1193              else: 
1194                  fn = Symbolic.expr2fun(str(val_q), **defs) 
1195                  if not self._direct_fail: 
1196                      fn_direct = Symbolic.expr2fun(str(val_q_direct), **defs) 
1197                      return ( (fn._args, fn), (fn_direct._args, fn_direct) ) 
1198                  else: 
1199                      return ( (fn._args, fn), (None, None) )

1200   
1201   


1202 -    def reset(self):


1203          """Delete current state based on given trajectory""" 
1204          self.traj = None 
1205          self.pts = None 
1206          self.times = None 
1207          self.focus_var = None 
1208          self.tau_inf_vals = {} 
1209          self.psi_vals = {} 
1210          self.Is = {} 
1211          self.pars = None

1212   
1213   


1214 -    def ensure(self):


1215          assert self.gen is not None, "Must have a valid generator attribute" 
1216          assert self.traj is not None, "Must provide trajectory attribute" 
1217          assert self.focus_var is not None, "Must provide focus_var attribute" 
1218          if self.pts is None: 
1219              self.pts = self.traj.sample() 
1220          if self.times is None: 
1221              self.times = self.pts.indepvararray 
1222          # refresh reference from generator 
1223          self.pars = self.gen.pars

1224   
1225   


1226 -    def calc_tau(self, target, pt):


1227          """Calculate tau of target variable directly from a given single point 
1228          containing only the state variables. 
1229          """ 
1230          assert not self._direct_fail, "Option not available" 
1231          ptFS = self._FScompatMap(pt) 
1232          try: 
1233              args, f = self.taus_direct[target] 
1234          except TypeError: 
1235              # entry in dict was None (can't broadcast) 
1236              return np.NaN 
1237          else: 
1238              try: 
1239                  return f(**common.filteredDict(ptFS, args)) 
1240              except: 
1241                  print f._args 
1242                  print f._call_spec 
1243                  raise

1244   
1245   


1246 -    def calc_inf(self, target, pt):


1247          """Calculate inf of target variable directly from a given single point 
1248          containing only the state variables. 
1249          """ 
1250          assert not self._direct_fail, "Option not available" 
1251          ptFS = self._FScompatMap(pt) 
1252          try: 
1253              args, f = self.infs_direct[target] 
1254          except TypeError: 
1255              # entry in dict was None (can't broadcast) 
1256              return np.NaN 
1257          else: 
1258              try: 
1259                  return f(**common.filteredDict(ptFS, args)) 
1260              except: 
1261                  print f._args 
1262                  print f._call_spec 
1263                  raise

1264   
1265   


1266 -    def calc_psi(self, target, pt, focus=None):


1267          """Calculate Psi of focus variable w.r.t. the target variable directly 
1268          from a given single point containing only the state variables. 
1269   
1270          If focus is None (default) then focus_var attribute is assumed. 
1271          """ 
1272          assert not self._direct_fail, "Option not available" 
1273          ptFS = self._FScompatMap(pt) 
1274          if focus is None: 
1275              focus = self.focus_var 
1276          try: 
1277              args, f = self.psis_direct[focus][target] 
1278          except TypeError: 
1279              # entry in dict was None (can't broadcast) 
1280              return np.NaN 
1281          except KeyError: 
1282              raise ValueError("Invalid focus or target variable") 
1283          else: 
1284              try: 
1285                  return f(**common.filteredDict(ptFS, args)) 
1286              except: 
1287                  print f._args 
1288                  print f._call_spec 
1289                  raise

1290   
1291   


1292 -    def calc_taus_infs(self, focus=False):


1293          """Calculate taus and infs from given trajectory. 
1294   
1295          If focus=True (default False), results are only returned for the 
1296          variable of focus given by focus_var attribute""" 
1297          self.ensure() 
1298          vals = {} 
1299          if focus: 
1300              vars = [self.focus_var] 
1301          else: 
1302              vars = self.taus.keys() 
1303          pts = self._FScompatMap(self.pts) 
1304  #        ptvals = dict(self.pts) 
1305  #        # permit access from external inputs or other autonomous functions 
1306  #        ptvals['_time_'] = pts['t'] 
1307  #        pts = Pointset(coorddict=ptvals, indepvararray=pts['t']) 
1308          for k in vars: 
1309              tau_info = self.taus[k] 
1310              if tau_info is None: 
1311                  vals['tau_'+k] = np.array([np.NaN for pt in pts]) 
1312              else: 
1313                  args, f = tau_info 
1314                  try: 
1315                      vals['tau_'+k] = np.array([f(**common.filteredDict(pt, args)) \ 
1316                                                 for pt in pts]) 
1317                  except: 
1318                      print f._args 
1319                      print f._call_spec 
1320                      raise 
1321              inf_info = self.infs[k] 
1322              if inf_info is None: 
1323                  vals['inf_'+k] = np.array([np.NaN for pt in pts]) 
1324              else: 
1325                  args, f = inf_info 
1326                  try: 
1327                      vals['inf_'+k] = np.array([f(**common.filteredDict(pt, args)) \ 
1328                                                 for pt in pts]) 
1329                  except: 
1330                      print f._args 
1331                      print f._call_spec 
1332                      raise 
1333          # we know that taus and infs have same set of keys -- the variable names 
1334          self.tau_inf_vals.update(vals)

1335   
1336   


1337 -    def calc_Is(self):


1338          """Calculate currents from given trajectory""" 
1339          raise NotImplementedError

1340   
1341   


1342 -    def calc_psis(self):


1343          """Calculate Psis from given trajectory""" 
1344          self.ensure() 
1345          FScMap = self._FScompatMap 
1346          fv = self.focus_var 
1347          if self.tau_inf_vals == {}: 
1348              self.calc_taus_infs(focus=True) 
1349          psi_defs = self.psis[fv] 
1350          if psi_defs is None: 
1351              self.psi_vals[fv] = None 
1352              return 
1353          psis = {} 
1354          all_var_inputs = self.gamma1[fv] + self.gamma2[fv] 
1355          pts = FScMap(self.pts) 
1356          ts = pts.indepvararray.copy() 
1357          ts.shape = (ts.shape[0],1) 
1358          c = pts.coordarray.T 
1359          all_coords = np.concatenate((c, ts), axis=1) 
1360          # how to reuse tau and inf values instead of calling functions repeatedly 
1361          #print "Use tau_v instead of tau_v(<args>) etc. for definition of psi function to take" 
1362          #print "advantage of pre-computed taus and infs" 
1363          for inp in all_var_inputs: 
1364              if inp not in psi_defs: 
1365                  continue 
1366              psi_info = psi_defs[inp] 
1367              if psi_info is None: 
1368                  psis[inp] = np.array([NaN]*len(pts)) 
1369              else: 
1370                  args, f = psi_info 
1371                  if 't' in args: 
1372                      tix = args.index('t') 
1373                      args.remove('t') 
1374                      arg_ixs = pts._map_names_to_ixs(args) 
1375                      arg_ixs.insert(tix, all_coords.shape[1]-1) 
1376                  else: 
1377                      arg_ixs = pts._map_names_to_ixs(args) 
1378                  try: 
1379                      psis[inp] = np.array([f(*val_array.flatten()) \ 
1380                                        for val_array in all_coords[:,[arg_ixs]]]) 
1381                  except: 
1382                      print f._args 
1383                      print f._call_spec 
1384                      raise 
1385          self.psi_vals[fv] = psis

1386   
1387   


1388 -    def make_pointsets(self):


1389          self.ensure() 
1390          fv = self.focus_var 
1391          assert self.psi_vals[fv] != {} 
1392          assert self.tau_inf_vals != {} 
1393          all_vals = {} 
1394          all_vals.update(self.tau_inf_vals) 
1395          base='psi_'+fv 
1396          for inp, psis in self.psi_vals[fv].items(): 
1397              all_vals[base+'_'+inp] = psis 
1398          self.psi_pts = Pointset(indepvararray=self.times, coorddict=all_vals)

1399   
1400   


1401 -    def calc_rankings(self, influence_type='psi'):


1402          self.ensure() 
1403          if not hasattr(self, influence_type+'_pts'): 
1404              raise "Call make_pointsets first" 
1405          else: 
1406              influence_pts = getattr(self, influence_type+'_pts') 
1407          varnames = influence_pts.coordnames 
1408          # filter out any non-influence_type coordinates 
1409          # (e.g. taus and infs that are included) 
1410          influence_pts = influence_pts[[v for v in varnames if v[:3]==influence_type]] 
1411          self.domscales = {influence_type: domscales(self.focus_var, self.pts, 
1412                                                      influence_pts, influence_type, 
1413                                                      self.tau_refs, self.psi_refs, 
1414                                                      self.opts)}

1415   
1416   


1417 -    def reduced_Vode_system(self, vars):


1418          """Doesn't allow fast vars to be algebraic - better solution is to use 
1419          ModelTransforms""" 
1420          gen = self.gen 
1421          name = "reduced_"+gen.funcspec._initargs['name']+"_"+"_".join(vars) 
1422          varspecs = {} 
1423          for xname in vars + gen.funcspec._initargs['auxvars']: 
1424              varspecs[xname] = gen.funcspec._initargs['varspecs'][xname] 
1425          sysargs = common.args(name=name, pars=gen.pars, 
1426                                ics=common.filteredDict(gen.initialconditions,vars), 
1427                                varspecs=varspecs, auxvars=gen.funcspec._initargs['auxvars'], 
1428                                fnspecs=gen.funcspec._initargs['fnspecs']) 
1429          nan_auxs = [xname for xname, val in gen.initialconditions.iteritems() if not np.isfinite(val)] 
1430          sysargs.pars.update(common.filteredDict(gen.initialconditions, vars+nan_auxs, neg=True)) 
1431          return Vode_ODEsystem(sysargs)

1432   
1433   
1434  #### HELPER FUNCTIONS 
1435   


1436 -def get_taus(pts, tau_names):


1437      """pts can be a Point or Pointset""" 
1438      # filter this way in case there are None entries in tau_names.values() 
1439      clist = [cname for cname in pts.coordnames if cname in tau_names.values()] 
1440      t = pts[clist] 
1441      t.mapNames(parseUtils.symbolMapClass(common.invertMap(tau_names))) 
1442      return t

1443   


1444 -def get_infs(pts, inf_names):


1445      """pts can be a Point or Pointset""" 
1446      # filter this way in case there are None entries in inf_names.values() 
1447      clist = [cname for cname in pts.coordnames if cname in inf_names.values()] 
1448      i = pts[clist] 
1449      i.mapNames(parseUtils.symbolMapClass(common.invertMap(inf_names))) 
1450      return i

1451   
1452   


1453 -def split_pts(pts, interval, reset_t0=None):


1454      """Return trajectory made up from pts argument restricted to the given 
1455      interval. 
1456   
1457      Use reset_t0 = T (float) to reset output trajectory t0 to T. 
1458      Default is to use native time frame of pts argument.""" 
1459      t0, t1 = interval 
1460      ix0 = pts.find(t0) 
1461      if not isinstance(ix0, int): 
1462          ix0 = ix0[0] 
1463      ix1 = pts.find(t1) 
1464      if not isinstance(ix1, int): 
1465          ix1 = ix1[1] 
1466      try: 
1467          vals = pts[ix0:ix1].coordarray.copy() 
1468      except ValueError: 
1469          print ix0, ix1 
1470          raise 
1471      ts = pts.indepvararray[ix0:ix1].copy() 
1472      if reset_t0 is not None: 
1473          ts += (reset_t0-ts[0]) 
1474      return numeric_to_traj(vals, 'split_%i_%i' % (ix0,ix1), 
1475                             pts.coordnames, 
1476                             indepvar=ts, discrete=False)

1477   
1478   


1479 -def find_epoch_period(epochs, verbose=False):


1480      cycle_len = None 
1481      ixs = [] 
1482      # don't start i at 0 b/c will be in middle of an epoch 
1483      for i, ep0 in enumerate(epochs[1:-1]): 
1484          if verbose: 
1485              print "start epoch = ", i+1 
1486          for j, ep in enumerate(epochs[i+1:]): 
1487              if verbose: 
1488                  print "end epoch =", i+j+1 
1489              try: 
1490                  res = [epochs[k]==epochs[k+j+1] for k in range(i,j)] 
1491              except IndexError: 
1492                  # too close to end, start next i 
1493                  continue 
1494              else: 
1495                  if verbose: 
1496                      print i, j, res 
1497              if np.all(res) and j > i+1: 
1498                  if verbose: 
1499                      print "Found period between epochs", i+1, j+2+i, " len=", j+1 
1500                  cycle_len = j+1 
1501                  ixs = [i+1, i+j+2] 
1502                  return cycle_len, ixs 
1503      return cycle_len, ixs

1504   
1505   


1506 -def split(ds_pt, thresh):


1507      """Split this grouping of values into sub-groups according to spectral 
1508      gaps at threshold thresh""" 
1509      sub_groups = [] 
1510      if ds_pt.relative_ratios: 
1511          spectral_gaps = spectral_gaps_relative 
1512      else: 
1513          spectral_gaps = spectral_gaps_absolute 
1514      sg = spectral_gaps(ds_pt.as_ratios, thresh) 
1515      for ix_range in partition_range((0, len(ds_pt.influences)), sg): 
1516          if ix_range[0] == ix_range[1]: 
1517              continue 
1518          sub_slice = slice(ix_range[0],ix_range[1]) 
1519          data = common.args() 
1520          data.influences = ds_pt.influences[sub_slice] 
1521          data.coordnames = ds_pt.coordnames[sub_slice] 
1522          sub_groups.append(data) 
1523      return sub_groups

1524   
1525   


1526 -def as_absolute_ratios(vals):


1527      """Express a single time point's values as ratios of the largest (>1). 
1528      first entry is ratio of largest to itself == 1. 
1529      """ 
1530      try: 
1531          return [1.0]+[vals[0]/v for v in vals[1:]] 
1532      except ZeroDivisionError: 
1533          rats = [1.0] 
1534          for i in range(1,len(vals)): 
1535              if vals[i] > 0: 
1536                  rats.append(vals[0]/vals[i]) 
1537              else: 
1538                  # don't include any more (0 is smallest possible influence) 
1539                  rats.append(numpy.inf) 
1540          return rats

1541   
1542   


1543 -def as_relative_ratios(vals):


1544      """Express a single time point's relative values as successive ratios (>1). 
1545      first entry is ratio of largest to itself == 1. 
1546      """ 
1547      try: 
1548          return [1.0]+[vals[i-1]/vals[i] \ 
1549                        for i in range(1,len(vals))] 
1550      except ZeroDivisionError: 
1551          rats = [1.0] 
1552          for i in range(1,len(vals)): 
1553              if vals[i] > 0: 
1554                  rats.append(vals[i-1]/vals[i]) 
1555              else: 
1556                  # don't include any more (0 is smallest possible influence) 
1557                  rats.append(numpy.inf) 
1558          return rats

1559   
1560   


1561 -def spectral_gaps_relative(as_rel_ratios, thresh):


1562      """List of indices of spectral gaps larger than thresh in size""" 
1563      # enumerating an array of Bools 
1564      return [i for i, is_gap in enumerate(np.asarray(as_rel_ratios) >= thresh) if is_gap]

1565   
1566   


1567 -def spectral_gaps_absolute(as_abs_ratios, thresh):


1568      """List of indices of spectral gaps larger than thresh in size""" 
1569      as_abs_ratios = np.asarray(as_abs_ratios) 
1570      gaps = [] 
1571      while True: 
1572          test = np.array(as_abs_ratios >= thresh, int) 
1573          if 1 in test: 
1574              # only makes sense to find gaps if there's a value > thresh 
1575              cutoff_ix = np.argmax(test) 
1576          else: 
1577              # only 0s present so no gaps remain 
1578              break 
1579          if gaps == []: 
1580              gaps.append(cutoff_ix) 
1581          else: 
1582              gaps.append(cutoff_ix+gaps[-1]) 
1583          as_abs_ratios = as_abs_ratios[cutoff_ix:] 
1584          if len(common.remain(as_abs_ratios, [np.inf])) <= 1: 
1585              break 
1586          else: 
1587              as_abs_ratios /= min(as_abs_ratios) 
1588      return gaps

1589   
1590   


1591 -def partition_range(range_tuple, split_ixs):


1592      # assumes everything is non-negative integer, and 
1593      # split_ixs is a list 
1594      if not range_tuple[0] < range_tuple[1]: 
1595          raise ValueError("Range tuple not in increasing order") 
1596      if not np.alltrue(ix in xrange(range_tuple[0],range_tuple[1]) \ 
1597                        for ix in split_ixs): 
1598          raise ValueError("Split indices out of range") 
1599      if range_tuple[0] in split_ixs or range_tuple[1] in split_ixs: 
1600          raise ValueError("Split indices out of range") 
1601      if split_ixs == []: 
1602          partition = [range_tuple] 
1603      else: 
1604          # make smallest index last 
1605          split_ixs.sort() 
1606          split_ixs.reverse() 
1607          end_ix = split_ixs.pop() 
1608          # end_ix not included in partition 
1609          partition = [(range_tuple[0],end_ix)] 
1610          while split_ixs != []: 
1611              # pop comes off end (smallest first) 
1612              next_split = (end_ix,split_ixs.pop()) 
1613              end_ix = next_split[1] 
1614              partition.append(next_split) 
1615          partition.append((end_ix,range_tuple[1])) 
1616      return partition

1617   
1618   


1619 -def normalized_psis(epochs, root, midpoint_only=True, small=1e-16):


1620      """Returns Pointset of normalized* psis based on contents of 'epochs' argument, 
1621      which would be returned from running da.domscales['psi'].calc_epochs() 
1622      where da is a domscales assistant class. 
1623   
1624        * Largest influence always normalized to 1 for every time point. 
1625      """ 
1626      def zero_to_small(v): 
1627          if v == 0: 
1628              return small 
1629          else: 
1630              return v

1631   
1632      inpnames = epochs[0].influences[0].coordnames 
1633      res = [] 
1634      ts = [] 
1635      if midpoint_only: 
1636          for ep in epochs: 
1637              numpts = len(ep.influences) 
1638              midpt = int(numpts/2) 
1639              ts.append(ep.influences['t'][midpt]) 
1640              repinf = ep.influences[midpt] 
1641              ranks = ep.ranks[midpt] 
1642              inv_ranks = common.invertMap(ranks) 
1643              vals_ord = np.take(repinf.coordarray, ranks) 
1644              ratios = np.array([1.0] + [zero_to_small(v)/vals_ord[0] for v in vals_ord[1:]]) 
1645              res.append(np.take(ratios, common.sortedDictValues(inv_ranks))) 
1646      else: 
1647          for ep in epochs: 
1648              numpts = len(ep.influences) 
1649              for nix in range(numpts): 
1650                  ts.append(ep.influences['t'][nix]) 
1651                  repinf = ep.influences[nix] 
1652                  ranks = ep.ranks[nix] 
1653                  inv_ranks = common.invertMap(ranks) 
1654                  vals_ord = np.take(repinf.coordarray, ranks) 
1655                  ratios = np.array([1.0] + [zero_to_small(v)/vals_ord[0] for v in vals_ord[1:]]) 
1656                  res.append(np.take(ratios, common.sortedDictValues(inv_ranks))) 
1657      coordnames = ['psi_%s_%s'%(root, i) for i in inpnames] 
1658      return Pointset(coordarray=np.array(res).T, indepvararray=ts, coordnames=coordnames) 
1659   
1660   
1661   


1662 -def show_epochs(eps):


1663      "Small utility to print more detailed information about epochs" 
1664      for i, ep in enumerate(eps): 
1665          print i, 
1666          ep.info()

1667   
1668   


1669 -def plot_psis(da, cols=None, do_vars=None, do_log=True, use_prefix=True):


1670      """da is a dssrt_assistant object. 
1671      cols is an optional dictionary mapping names of Psi entries to specific color/style character codes. 
1672      Pass do_vars list of Psi names to restrict, otherwise all will be plotted. 
1673      Option to plot on vertical log scale. 
1674   
1675      Option to switch off 'psi_' + da's focus_var as prefix for coordnames. 
1676   
1677      Requires matplotlib. 
1678      """ 
1679      from pylab import plot 
1680      pts=da.psi_pts 
1681      if use_prefix: 
1682          root = 'psi_'+da.focus_var+'_' 
1683      else: 
1684          root = '' 
1685      ts = pts['t'] 
1686      if do_vars is None: 
1687          do_vars = [c[len(root):] for c in pts.coordnames if root in c] 
1688      if cols is None: 
1689          colvals = ['g', 'r', 'k', 'b', 'c', 'y', 'm'] 
1690          styles = ['-', ':', '--'] 
1691          cols = [] 
1692          for s in styles: 
1693              for c in colvals: 
1694                  cols.append(c+s) 
1695      if len(do_vars) > len(cols): 
1696          raise ValueError("Max number of permitted variables for these colors/styles is %i"%len(cols)) 
1697      print "Color scheme:" 
1698      if do_log: 
1699          for i, v in enumerate(do_vars): 
1700              if root+v in pts.coordnames: 
1701                  print " ", v, cols[i] 
1702                  plot(ts, np.log(pts[root+v]), cols[i]) 
1703      else: 
1704          for i, v in enumerate(do_vars): 
1705              if root+v in pts.coordnames: 
1706                  print " ", v, cols[i] 
1707                  plot(ts, pts[root+v], cols[i])

1708   
1709  # --------------------------------------------------------------------------- 
1710  # Sequence comparison code 
1711   
1712  MATCH = 'match' 
1713  MISMATCH = 'mismatch' 
1714  CLOSE = 'close' 
1715  FAR = 'far' 
1716  VCLOSE = 'vclose' 
1717  VFAR = 'vfar' 
1718  GAP = 'gap' 
1719  APPROX = 'approx' 
1720   


1721 -class Scorer(object):


1722 -    def __init__(self, alphabet):


1723          self.match = None 
1724          self.approx = None 
1725          self.mismatch = None 
1726          self.gap = None 
1727          self.extension = None 
1728          self.alphabet = alphabet 
1729          pairs = utils.cartesianProduct(alphabet.all_vars, alphabet.all_vars) 
1730          self.score_dict = {}.fromkeys(pairs, None)

1731   


1732 -    def __setitem__(self, k, v):


1733          if k in self.score_dict: 
1734              self.score_dict[k] = v 
1735              # also symmetric value 
1736              a, b = k 
1737              self.score_dict[(b, a)] = v 
1738          else: 
1739              raise KeyError("Invalid key %s"%k)

1740   


1741 -    def __getitem__(self, k):


1742          return getattr(self, self.score_dict[k])

1743   
1744   


1745 -class EpochSeqScorer(Scorer):


1746 -    def __init__(self, alphabet):


1747          Scorer.__init__(self, alphabet) 
1748          self.match = 5 
1749          self.gap = -2  # unused? 
1750          self.mismatch = -4 
1751          self.extension = -1 
1752   
1753          fast = alphabet.fast 
1754          slow = alphabet.slow 
1755   
1756          s1 = Scorer(alphabet) 
1757          s1.match = 5 
1758          s1.vclose = 4 
1759          s1.close = 2 
1760          s1.far = -1 
1761          s1.vfar = -3 
1762          s1.mismatch = -5 
1763          s1.gap = -2 
1764          s1.extension = -1 
1765   
1766          s2 = Scorer(alphabet) 
1767          s2.gap = 1 
1768          s2.mismatch = 0 
1769          s2.far = 2 
1770          s2.vfar = 1 
1771          s2.close = 3 
1772          s2.vclose = 4 
1773          s2.match = 6 
1774          s2.extension = 0 
1775   
1776          for a in alphabet.dynamic_vars: 
1777              s1[(a,a)] = MATCH 
1778              s1[(fast(a),fast(a))] = MATCH 
1779              s1[(slow(a),slow(a))] = MATCH 
1780              s1[(fast(a),a)] = VCLOSE 
1781              s1[(slow(a),a)] = VCLOSE 
1782              s1[(slow(a),fast(a))] = CLOSE 
1783   
1784              # same 
1785              s2[(a,a)] = MATCH 
1786              s2[(fast(a),fast(a))] = MATCH 
1787              s2[(slow(a),slow(a))] = MATCH 
1788              s2[(fast(a),a)] = VCLOSE 
1789              s2[(slow(a),a)] = VCLOSE 
1790              s2[(slow(a),fast(a))] = CLOSE 
1791   
1792          s1[('_','_')] = CLOSE 
1793          s2[('_','_')] = MATCH 
1794   
1795          for a in alphabet.non_dynamic_vars: 
1796              s1[(a,a)] = MATCH 
1797              s1[(a,'_')] = CLOSE 
1798              s2[(a,a)] = MATCH #VCLOSE 
1799              s2[(a,'_')] = CLOSE 
1800   
1801          for k, v in s1.score_dict.iteritems(): 
1802              if v is None: 
1803                  d_common = len(common.intersect(alphabet.dynamic_vars, strip_speed(k))) 
1804          #        s.score_dict[k] = MISMATCH 
1805                  if (k[0] == '_' or k[1] == '_') and k[0] != k[1]: 
1806                      if d_common > 0: 
1807                          s1.score_dict[k] = MISMATCH 
1808                          s2.score_dict[k] = VFAR 
1809                      else: 
1810                          s1.score_dict[k] = MISMATCH 
1811                          s2.score_dict[k] = VFAR 
1812                  elif d_common > 0: 
1813                      s1.score_dict[k] = MISMATCH 
1814                      s2.score_dict[k] = VFAR #MISMATCH 
1815                  else: 
1816                      s1.score_dict[k] = MISMATCH 
1817                      s2.score_dict[k] = CLOSE 
1818   
1819          self.s1 = s1 
1820          self.s2 = s2 
1821   
1822          self.weight_unordered = 0.35 
1823          self.weight_s1 = 0.2 
1824          self.weight_s2 = 0.45

1825   


1826 -    def _validate_weights(self):


1827          assert self.weight_s1 + self.weight_s2 + self.weight_unordered == 1

1828   


1829 -    def __getitem__(self, k):


1830          return self.scorer(k[0], k[1])*self.match

1831   
1832   


1833 -    def scorer(self, a, b):


1834          self._validate_weights() 
1835          s1 = self.s1 
1836          s2 = self.s2 
1837          minlen = min(len(a),len(b)) 
1838          maxlen = max(len(a),len(b)) 
1839          a_un = [] 
1840          b_un = [] 
1841          a_strip = strip_speed(a) 
1842          b_strip = strip_speed(b) 
1843          seen_cs = [] 
1844          for c in utils.union(a, b): 
1845              cs = strip_speed([c])[0] 
1846              if cs in seen_cs: 
1847                  continue 
1848              else: 
1849                  seen_cs.append(cs) 
1850              try: 
1851                  ixb = b_strip.index(cs) 
1852              except ValueError: 
1853                  pass 
1854              else: 
1855                  try: 
1856                      ixa = a_strip.index(cs) 
1857                  except ValueError: 
1858                      pass 
1859                  else: 
1860                      a_un.append(a[ixa]) 
1861                      b_un.append(b[ixb]) 
1862          a_un = a_un + common.remain(a, a_un) 
1863          b_un = b_un + common.remain(b, b_un) 
1864          a_dyn = only_dynamic(pad(a, maxlen), s1.alphabet.dynamic_vars) 
1865          b_dyn = only_dynamic(pad(b, maxlen), s1.alphabet.dynamic_vars) 
1866          #unordered_sim = swdist(pad(a_un, maxlen), pad(b_un, maxlen), s) 
1867          unordered_sim = sum([s2[(pad(a_un, maxlen)[i], pad(b_un, maxlen)[i])] for i in range(maxlen)])/(maxlen*s2.match) 
1868          #print pad(a_un, maxlen), pad(b_un, maxlen), [s2[(pad(a_un, maxlen)[i], pad(b_un, maxlen)[i])] for i in range(maxlen)] 
1869          #print "\n ", unordered_sim, swdist(a_dyn, b_dyn, s1), swdist(a, b, s2) 
1870          return (self.weight_unordered*unordered_sim + \ 
1871                  self.weight_s1*swdist(a_dyn, b_dyn, s1) + \ 
1872                  self.weight_s2*swdist(a, b, s2))

1873          #return (unordered_sim*swdist(a_dyn, b_dyn, s1)*swdist(a,b,s2))**0.33333333333 
1874   
1875   


1876 -def get_symbol_sequence(epoch_list, get_actives=True, get_modulatory=False):


1877      seq = [] 
1878      for ep in epoch_list: 
1879          a, m = ep.seq_str() 
1880          if get_actives: 
1881              if get_modulatory: 
1882                  l = (a, m) 
1883              else: 
1884                  l = a 
1885          else: 
1886              if get_modulatory: 
1887                  l = m 
1888              else: 
1889                  raise "Must specify at least one type of output" 
1890          seq.append(l) 
1891      return seq

1892   


1893 -class VarAlphabet(object):


1894 -    def __init__(self, dynamic, non_dynamic):


1895          self.dynamic_vars = dynamic 
1896          self.non_dynamic_vars = non_dynamic 
1897          alphabet = dynamic + non_dynamic 
1898          for speed in ['F', 'S']: 
1899              alphabet.extend([v+'[%s]'%speed for v in dynamic]) 
1900          self.all_vars = alphabet + ['_']

1901   


1902 -    def fast(self, a):


1903          if a in self.dynamic_vars: 
1904              return a+'[F]' 
1905          else: 
1906              raise ValueError("%s is not a dynamic variable" % a)

1907   


1908 -    def slow(self, a):


1909          if a in self.dynamic_vars: 
1910              return a+'[S]' 
1911          else: 
1912              raise ValueError("%s is not a dynamic variable" % a)

1913   
1914  # INTERNAL DSSRT SEQUENCE UTILITIES 


1915 -def strip_speed(seq):


1916      res = [] 
1917      for a in seq: 
1918          if a[-1] == ']': 
1919              res.append(a[:-3]) 
1920          else: 
1921              res.append(a) 
1922      return res

1923   


1924 -def pad(seq, n):


1925      m = len(seq) 
1926      if m > n: 
1927          raise ValueError 
1928      elif m < n: 
1929          return seq + ['_']*(n-m) 
1930      else: 
1931          return seq

1932   


1933 -def only_dynamic(seq, dynamic_vars):


1934      res = [] 
1935      for a in seq: 
1936          if strip_speed([a])[0] in dynamic_vars: 
1937              res.append(a) 
1938          else: 
1939              res.append('_') 
1940      return res

1941   
1942   


1943 -def comp_seqs(seq1, seq2, scorer):


1944      """Based on code by Gergely Szollosi""" 
1945      rows=len(seq1)+1 
1946      cols=len(seq2)+1 
1947      a=np.zeros((rows,cols),float) 
1948      if isinstance(seq1, list): 
1949          blank = [['_']] 
1950      else: 
1951          blank = ['_'] 
1952      for i in range(1,rows): 
1953          for j in range(1,cols): 
1954              # Dynamic programing -- aka. divide and conquer: 
1955              # Since gap penalties are linear in gap size 
1956              # the score of an alignmet of length l only depends on the 
1957              # the l-th characters in the alignment (match - mismatch - gap) 
1958              # and the score of the one shorter (l-1) alignment, 
1959              # i.e. we can calculate how to extend an arbritary alignment 
1960              # soley based on the previous score value. 
1961              choice1 = a[i-1, j-1] + scorer[(seq1[i-1], seq2[j-1])] 
1962              choice2 = a[i-1, j] + scorer.gap 
1963              choice3 = a[i, j-1] + scorer.gap 
1964              a[i, j] = max(choice1, choice2, choice3) 
1965   
1966      aseq1 = [] 
1967      aseq2 = [] 
1968      #We reconstruct the alignment into aseq1 and aseq2, 
1969      i = len(seq1) 
1970      j = len(seq2) 
1971      while i>0 and j>0: 
1972          #by performing a traceback of how the matrix was filled out above, 
1973          #i.e. we find a shortest path from a[n,m] to a[0,0] 
1974          score = a[i, j] 
1975          score_diag = a[i-1, j-1] 
1976          score_up = a[i, j-1] 
1977          score_left = a[i-1, j] 
1978          if score == score_diag + scorer[(seq1[i-1], seq2[j-1])]: 
1979              aseq1 = [seq1[i-1]] + aseq1 
1980              aseq2 = [seq2[j-1]] + aseq2 
1981              i -= 1 
1982              j -= 1 
1983          elif score == score_left + scorer.gap: 
1984              aseq1 = [seq1[i-1]] + aseq1 
1985              aseq2 = blank + aseq2 
1986              i -= 1 
1987          elif score == score_up + scorer.gap: 
1988              aseq1 = blank + aseq1 
1989              aseq2 = [seq2[j-1]] + aseq2 
1990              j -= 1 
1991          else: 
1992              #should never get here.. 
1993              raise RuntimeError 
1994      while i>0: 
1995          #If we hit j==0 before i==0 we keep going in i. 
1996          aseq1 = [seq1[i-1]] + aseq1 
1997          aseq2 = blank + aseq2 
1998          i -= 1 
1999   
2000      while j>0: 
2001          #If we hit i==0 before i==0 we keep going in j. 
2002          aseq1 = blank + aseq1 
2003          aseq2 = [seq2[j-1]] + aseq2 
2004          j -= 1 
2005   
2006      final_score = swdist(aseq1, aseq2, scorer) 
2007      #g = make_graph(seq1, seq2, a, rows, cols, scorer) 
2008      return (aseq1, aseq2), final_score

2009   
2010   
2011  ##def make_graph(seq1, seq2, a, rows, cols, scorer): 
2012  ##    #the simplest way is to make a graph of the possible constructions of the values in a 
2013  ##    graph={} 
2014  ##    for i in range(1,cols)[::-1]: 
2015  ##        graph[(i,0)] = [(i-1,0)] 
2016  ##        graph[(0,i)] = [(0,i-1)] 
2017  ##        for j in range(1,cols)[::-1]: 
2018  ##            graph[(i,j)]=[] 
2019  ##            score = a[i, j] 
2020  ##            score_diag = a[i-1, j-1] 
2021  ##            score_up = a[i, j-1] 
2022  ##            score_left = a[i-1, j] 
2023  ##            if score == score_diag + scorer[(seq1[i-1], seq2[j-1])]: 
2024  ##                graph[(i,j)] += [(i-1,j-1)] 
2025  ##            if score == score_left + scorer.gap: 
2026  ##                graph[(i,j)] += [(i-1,j)] 
2027  ##            if score == score_up + scorer.gap: 
2028  ##                graph[(i,j)] += [(i,j-1)] 
2029  ##    return graph 
2030   


2031 -def swdist(str1, str2, scorer, common_divisor='longest', min_threshold=None):


2032      """Return approximate string comparator measure (between 0.0 and 1.0) 
2033         using the Smith-Waterman distance. 
2034   
2035      USAGE: 
2036        score = swdist(str1, str2, scorer, common_divisor, min_threshold) 
2037   
2038      ARGUMENTS: 
2039        str1            The first sequence 
2040        str2            The second sequence 
2041        scorer          scorer object instance to compare two symbols 
2042        common_divisor  Method of how to calculate the divisor, it can be set to 
2043                        'average','shortest', or 'longest' , and is calculated 
2044                        according to the lengths of the two input strings 
2045        min_threshold   Minimum threshold between 0 and 1 
2046   
2047      DESCRIPTION: 
2048        Smith-Waterman distance is commonly used in biological sequence alignment. 
2049   
2050        Scores for matches, misses, gap and extension penalties are set to values 
2051        described in the scorer 
2052      """ 
2053   
2054      # Quick check if the strings are empty or the same - - - - - - - - - - - - - 
2055      # 
2056   
2057      n = len(str1) 
2058      m = len(str2) 
2059   
2060      if n*m == 0: 
2061          return 0.0 
2062      elif str1 == str2: 
2063          return 1.0 
2064   
2065      # Scores used for Smith-Waterman algorithm - - - - - - - - - - - - - - - - - 
2066      # 
2067  #    match_score =       5 
2068  #    approx_score =      2 
2069  #    mismatch_score =   -5 
2070  #    gap_penalty =       5 
2071  #    extension_penalty = 1 
2072   
2073      # Calculate the divisor - - - - - - - - - - - - - - - - - - - - - - - - - - - 
2074      # 
2075      if (common_divisor not in ['average','shortest','longest']): 
2076          raise ValueError('Illegal value for common divisor: %s' % \ 
2077                           (common_divisor)) 
2078   
2079      if common_divisor == 'average': 
2080          divisor = 0.5*(n+m)*scorer.match  # Average maximum score 
2081      elif common_divisor == 'shortest': 
2082          divisor = min(n,m)*scorer.match 
2083      else:  # Longest 
2084          divisor = max(n,m)*scorer.match 
2085   
2086      best_score = 0  # Keep the best score while calculating table 
2087   
2088      d = np.zeros((n+1,m+1),float)  # distance matrix 
2089   
2090      for i in range(1,n+1): 
2091          for j in range(1,m+1): 
2092   
2093              match = d[i-1, j-1] + scorer[(str1[i-1], str2[j-1])] 
2094   
2095              insert = 0 
2096              for k in range(1,i): 
2097                  score = d[i-k, j] + scorer.gap + k*scorer.extension 
2098                  insert = max(insert, score) 
2099   
2100              delete = 0 
2101              for l in range(1,j): 
2102                  score = d[i, j-l] + scorer.gap + l*scorer.extension 
2103                  delete = max(delete, score) 
2104   
2105              d[i, j] = max(match, insert, delete, 0) 
2106              best_score = max(d[i, j], best_score) 
2107   
2108      # best_score can be min(len(str1),len(str2))*match_score (if one string is 
2109      # a sub-string of the other string). 
2110      # 
2111      # The lower best_score the less similar the sequences are. 
2112      # 
2113      w = float(best_score) / float(divisor) 
2114      assert (w >= 0.0) and (w <= 1.0), 'Similarity weight outside 0-1: %f' % (w) 
2115      return w

2116   
2117   


2118 -def editdist_edits(str1, str2):


2119      """Return approximate string comparator measure (between 0.0 and 1.0) 
2120       using the edit (or Levenshtein) distance as well as a triplet with the 
2121       counts of the actual edits (inserts, deletes and substitutions). 
2122   
2123    USAGE: 
2124      score, edit_counts = editdist_edits(str1, str2) 
2125   
2126    ARGUMENTS: 
2127      str1           The first seq 
2128      str2           The second seq 
2129   
2130    DESCRIPTION: 
2131      The edit distance is the minimal number of insertions, deletions and 
2132      substitutions needed to make two strings equal. 
2133   
2134      edit_counts  is a list with three elements that contain the number of 
2135                   inserts, deletes and substitutions that were needed to convert 
2136                   str1 into str2. 
2137   
2138      For more information on the modified Soundex see: 
2139      - http://www.nist.gov/dads/HTML/editdistance.html 
2140    """ 
2141   
2142      # Check if the strings are empty or the same - - - - - - - - - - - - - - - - 
2143      # 
2144      n = len(str1) 
2145      m = len(str2) 
2146   
2147      if n + m == 0: 
2148          return 0.0, [0,0,0] 
2149   
2150      elif n*m == 0: 
2151          if n == 0: 
2152              return 0.0, [m,0,0]    # Inserts needed to get from empty to str1 
2153          else: 
2154              return 0.0, [0,n,0,0]  # Deletes nedded to get from str2 to empty 
2155   
2156      elif str1 == str2: 
2157          return 1.0, [0,0,0] 
2158   
2159      d = []  # Table with the full distance matrix 
2160   
2161      current = range(n+1) 
2162      d.append(current) 
2163   
2164      for i in range(1,m+1): 
2165   
2166          previous = current 
2167          current =  [i]+n*[0] 
2168          str2char = str2[i-1] 
2169   
2170          for j in range(1,n+1): 
2171              substitute = previous[j-1] 
2172              if (str1[j-1] != str2char): 
2173                  substitute += 1 
2174   
2175              # Get minimum of insert, delete and substitute 
2176              # 
2177              current[j] = min(previous[j]+1, current[j-1]+1, substitute) 
2178   
2179          d.append(current) 
2180   
2181      # Count the number of edits that were needed - - - - - - - - - - - - - - - - 
2182      # 
2183      num_edits = [0,0,0]  # Number of Inserts, deletes and substitutions 
2184   
2185      d_curr = d[m][n]  # Start with final position in table 
2186      j = n 
2187      i = m 
2188   
2189      while (d_curr > 0): 
2190          if (d[i-1][j-1]+1 == d_curr):  # Substitution 
2191              i -= 1 
2192              j -= 1 
2193              num_edits[2] += 1 
2194          elif (d[i-1][j]+1 == d_curr):  # Delete 
2195              i -= 1 
2196              num_edits[1] += 1 
2197          elif (d[i][j-1]+1 == d_curr):  # Insert 
2198              j -= 1 
2199              num_edits[0] += 1 
2200   
2201          else:  # Current position not larger than any of the previous positions 
2202              if (d[i-1][j-1] == d_curr): 
2203                  i -= 1 
2204                  j -= 1 
2205              elif (d[i-1][j] == d_curr): 
2206                  i -= 1 
2207              elif (d[i][j-1] == d_curr): 
2208                  j -= 1 
2209          d_curr = d[i][j]  # Update current position in table 
2210   
2211      w = 1.0 - float(d[m][n]) / float(max(n,m)) 
2212      assert (w >= 0.0) and (w <= 1.0), 'Similarity weight outside 0-1: %f' % (w) 
2213   
2214      return w, num_edits

2215   
2216   
2217   
2218  JARO_MARKER_CHAR = '*' 
2219   


2220 -def jaro(str1, str2, min_threshold=None):


2221      """Return approximate string comparator measure (between 0.0 and 1.0) 
2222   
2223    USAGE: 
2224      score = jaro(str1, str2, min_threshold) 
2225   
2226    ARGUMENTS: 
2227      str1           The first string 
2228      str2           The second string 
2229      min_threshold  Minimum threshold between 0 and 1 (currently not used) 
2230   
2231    DESCRIPTION: 
2232      As desribed in 'An Application of the Fellegi-Sunter Model of 
2233      Record Linkage to the 1990 U.S. Decennial Census' by William E. Winkler 
2234      and Yves Thibaudeau. 
2235    """ 
2236   
2237      # Quick check if the strings are empty or the same - - - - - - - - - - - - - 
2238      # 
2239      if (str1 == '') or (str2 == ''): 
2240          return 0.0 
2241      elif (str1 == str2): 
2242          return 1.0 
2243   
2244      len1 = len(str1) 
2245      len2 = len(str2) 
2246   
2247      halflen = max(len1,len2) / 2 + 1 
2248   
2249      ass1 = ''  # Characters assigned in str1 
2250      ass2 = ''  # Characters assigned in str2 
2251   
2252      workstr1 = str1  # Copy of original string 
2253      workstr2 = str2 
2254   
2255      common1 = 0  # Number of common characters 
2256      common2 = 0 
2257   
2258      # Analyse the first string  - - - - - - - - - - - - - - - - - - - - - - - - - 
2259      # 
2260      for i in range(len1): 
2261          start = max(0,i-halflen) 
2262          end   = min(i+halflen+1,len2) 
2263          index = workstr2.find(str1[i],start,end) 
2264          if (index > -1):  # Found common character 
2265              common1 += 1 
2266              ass1 = ass1 + str1[i] 
2267              workstr2 = workstr2[:index]+JARO_MARKER_CHAR+workstr2[index+1:] 
2268   
2269      # Analyse the second string - - - - - - - - - - - - - - - - - - - - - - - - - 
2270      # 
2271      for i in range(len2): 
2272          start = max(0,i-halflen) 
2273          end   = min(i+halflen+1,len1) 
2274          index = workstr1.find(str2[i],start,end) 
2275          if (index > -1):  # Found common character 
2276              common2 += 1 
2277              ass2 = ass2 + str2[i] 
2278              workstr1 = workstr1[:index]+JARO_MARKER_CHAR+workstr1[index+1:] 
2279   
2280      if (common1 != common2): 
2281          print 'Jaro: Wrong common values for strings "%s" and "%s"' % \ 
2282                           (str1, str2) + ', common1: %i, common2: %i' % (common1, common2) + \ 
2283                           ', common should be the same.' 
2284          common1 = float(common1+common2) / 2.0  ##### This is just a fix ##### 
2285   
2286      if (common1 == 0): 
2287          return 0.0 
2288   
2289      # Compute number of transpositions  - - - - - - - - - - - - - - - - - - - - - 
2290      # 
2291      transposition = 0 
2292      for i in range(len(ass1)): 
2293          if (ass1[i] != ass2[i]): 
2294              transposition += 1 
2295      transposition = transposition / 2.0 
2296   
2297      common1 = float(common1) 
2298      w = 1./3.*(common1 / float(len1) + common1 / float(len2) + \ 
2299                 (common1-transposition) / common1) 
2300   
2301      assert (w >= 0.0) and (w <= 1.0), 'Similarity weight outside 0-1: %f' % (w) 
2302   
2303      return w

2304   
2305   


2306 -def tabulate_epoch_seqs(epseq1, epseq2):


2307      """Create a text table of epochs""" 
2308      str_table = [] 
2309      assert len(epseq1) == len(epseq2) 
2310      for i in range(len(epseq1)): 
2311          eps1 = epseq1[i] 
2312          eps2 = epseq2[i] 
2313          str_table.append([",".join(eps1), ",".join(eps2)]) 
2314      print indent(str_table)

2315   


2316 -def indent(rows, hasHeader=False, headerChar='-', delim=' | ', justify='left', 
2317             separateRows=False, prefix='', postfix='', wrapfunc=lambda x:x):


2318      """Indents a table by column. 
2319         - rows: A sequence of sequences of items, one sequence per row. 
2320         - hasHeader: True if the first row consists of the columns' names. 
2321         - headerChar: Character to be used for the row separator line 
2322           (if hasHeader==True or separateRows==True). 
2323         - delim: The column delimiter. 
2324         - justify: Determines how are data justified in their column. 
2325           Valid values are 'left','right' and 'center'. 
2326         - separateRows: True if rows are to be separated by a line 
2327           of 'headerChar's. 
2328         - prefix: A string prepended to each printed row. 
2329         - postfix: A string appended to each printed row. 
2330         - wrapfunc: A function f(text) for wrapping text; each element in 
2331           the table is first wrapped by this function.""" 
2332      # closure for breaking logical rows to physical, using wrapfunc 
2333      def rowWrapper(row): 
2334          newRows = [wrapfunc(item).split('\n') for item in row] 
2335          return [[substr or '' for substr in item] for item in map(None,*newRows)]

2336      # break each logical row into one or more physical ones 
2337      logicalRows = [rowWrapper(row) for row in rows] 
2338      # columns of physical rows 
2339      columns = map(None,*reduce(operator.add,logicalRows)) 
2340      # get the maximum of each column by the string length of its items 
2341      maxWidths = [max([len(str(item)) for item in column]) for column in columns] 
2342      rowSeparator = headerChar * (len(prefix) + len(postfix) + sum(maxWidths) + \ 
2343                                   len(delim)*(len(maxWidths)-1)) 
2344      # select the appropriate justify method 
2345      justify = {'center':str.center, 'right':str.rjust, 'left':str.ljust}[justify.lower()] 
2346      output=cStringIO.StringIO() 
2347      if separateRows: print >> output, rowSeparator 
2348      for physicalRows in logicalRows: 
2349          for row in physicalRows: 
2350              print >> output, \ 
2351                  prefix \ 
2352                  + delim.join([justify(str(item),width) for (item,width) in zip(row,maxWidths)]) \ 
2353                  + postfix 
2354          if separateRows or hasHeader: print >> output, rowSeparator; hasHeader=False 
2355      return output.getvalue() 
2356   
2357   
2358   
2359  # --------------------------------------------------------------------------- 
2360   
2361  if __name__ == '__main__': 
2362      # Tests 
2363      ep1=common.args(actives=['x','y'],modulatory=['z','w'],sigma=2,relative_ratios=True) 
2364      res1 = transition_psi(ep1, Point({'x': 1, 'y': 0.499, 'z': 0.2, 'w': 0.1}), 0.01) 
2365      assert res1 == ('leave', 'y') 
2366      res2 = transition_psi(ep1, Point({'x': 1, 'y': 0.601, 'z': 0.3, 'w': 0.1}), 0.01) 
2367      assert res2 == ('join', 'z') 
2368      ep2=common.args(actives=['x'],modulatory=['z','w'],sigma=2,relative_ratios=True) 
2369      res3 = transition_psi(ep2, Point({'x': 1, 'z': 0.1, 'w': 0.501}), 0.01) 
2370      assert res3 == ('join', 'w') 
2371      try: 
2372          res4 = transition_psi(ep2, Point({'x': 1, 'z': 2, 'w': 0.1}), 0.01) 
2373      except PyDSTool_ValueError: 
2374          pass 
2375      else: 
2376          raise AssertionError 
2377   
2378      ep1=common.args(actives=['x','y'],modulatory=['z','w'],sigma=2,relative_ratios=False) 
2379      res1 = transition_psi(ep1, Point({'x': 1, 'y': 0.499, 'z': 0.2, 'w': 0.1}), 0.01) 
2380      assert res1 == ('leave', 'y') 
2381      res2 = transition_psi(ep1, Point({'x': 1, 'y': 0.601, 'z': 0.499, 'w': 0.1}), 0.01) 
2382      assert res2 == ('join', 'z') 
2383      ep2=common.args(actives=['x'],modulatory=['z','w'],sigma=2,relative_ratios=False) 
2384      res3 = transition_psi(ep2, Point({'x': 1, 'z': 0.1, 'w': 0.501}), 0.01) 
2385      assert res3 == ('join', 'w') 
2386      try: 
2387          res4 = transition_psi(ep2, Point({'x': 1, 'z': 2, 'w': 0.1}), 0.01) 
2388      except PyDSTool_ValueError: 
2389          pass 
2390      else: 
2391          raise AssertionError 
2392      ep3=common.args(actives=['x','y'],modulatory=[],sigma=2,relative_ratios=False) 
2393      res5 = transition_psi(ep3, Point({'x': 1, 'y': 0.501}), 0.01) 
2394      assert res5 == ('leave', 'y') 
2395   
2396      ep1=common.args(fast=['x','y'],slow=['w','z'],order1=['v'],gamma=2) 
2397      res1 = transition_tau(ep1, Point({'x': 0.3, 'y': 0.2, 'v':1, 'w': 2.005, 'z': 3}), 0.01) 
2398      assert res1 == ('slow_leave', 'w') 
2399
```

  


| Home | Trees | Indices | Help | PyDSTool |
| --- | --- | --- | --- | --- |

|  |  |
| --- | --- |
| Generated by Epydoc 3.0.1 on Fri May 4 15:24:24 2012 | http://epydoc.sourceforge.net |
